# GABRP Mediates GABA‐A Receptor to Shape Tumor Immunosuppressive Microenvironment and Promote Tumor Immune Escape and Corresponding Targeted Therapy

**DOI:** 10.1002/cam4.70946

**Published:** 2025-06-03

**Authors:** Wu Cen, Genyuan Fu, Xiaoyu Wang, Ruting Wei, Xingwang Zhou, Wei Teng, Yuanguo Ling, Jiaze Tang, Zhongan Wang, Liangzhao Chu

**Affiliations:** ^1^ Xingyi People's Hospital Affiliated to Guizhou Medical University Xingyi Guizhou Province China; ^2^ The Affiliated Hospital of Guizhou Medical University Guiyang Guizhou Province China

**Keywords:** cancer cell, GABA receptor, GABRP gene, treatment, tumor microenvironment

## Abstract

**Background:**

Tumor immune evasion mediated by the immunosuppressive tumor microenvironment (TME) remains a major obstacle in cancer therapy. The γ‐aminobutyric acid receptor π subunit (GABRP) is aberrantly expressed in cancers, but its role in immune evasion is poorly defined.

**Objective:**

To elucidate the mechanism of GABRP‐driven TME remodeling and evaluate its therapeutic potential.

**Materials and Methods:**

Pan‐cancer bioinformatics analysis (TCGA, GEPIA2, cBioPortal) assessed GABRP expression, survival associations, and immune infiltration across 33 cancers. Functional studies included GABRP knockdown in glioma cells (U87/U251) via lentiviral RNAi, proliferation/migration assays (CCK‐8, scratch test), and pathway analysis. Subcutaneous xenografts in BALB/c‐nu mice evaluated the GABA_A inhibitor Amentoflavone. Immune profiling utilized ssGSEA and TIMER.

**Results:**

GABRP was overexpressed in gliomas and other cancers (breast, gastric), correlating with poor prognosis (HR = 1.8, *p* = 0.008) and enriched immunosuppressive cells (Tregs, M2 macrophages). Knockdown suppressed proliferation (IC50↓42%), migration (> 50% delay, *p* < 0.01), and PI3K/AKT signaling. Amentoflavone reduced tumor volume by 68% (*p* < 0.001) and reversed GABA‐mediated T cell inhibition.

**Discussion:**

GABRP promotes immune evasion via GABA overproduction, recruiting Tregs/M2 macrophages to establish an immunosuppressive TME. Targeting GABRP or GABA signaling (e.g., Amentoflavone) restores antitumor immunity. Limitations include cohort size and tissue‐specific heterogeneity.

**Conclusion:**

GABRP is a key regulator of tumor immunosuppression. Dual strategies—blocking GABRP expression or GABA signaling—offer novel therapeutic avenues. Clinical validation is needed to advance precision oncology.

## Graphical abstract

Beyond its well‐established role in suppressing neuronal excitability, GABA (γ‐aminobutyric acid) has been shown to inhibit immune cell functions. Cancer cells exploit GABA signaling to modulate the tumor microenvironment and impair normal immune cell activities, thereby facilitating immune evasion. Emerging evidence suggests that targeted suppression of GABRP gene expression in cancer cells or pharmacological intervention with GABA‐A receptor inhibitors could enable effective and precise immunotherapeutic strategies against cancer.

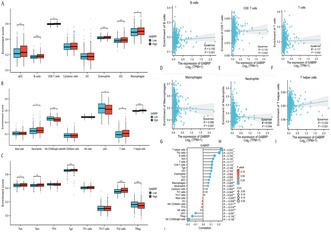

## 1 Introduction

For millennia, cancer has consistently ranked as one of the most pressing health issues globally [1]. It is a common disease with persistently high mortality rates, and traditional treatment methods often yield limited efficacy, imposing a significant psychological and economic burden on patients and their families [2]. The World Health Organization (WHO) reports that there are approximately 20 million new cancer cases worldwide each year, leading to around 9 million deaths annually [3]. This year, the American Cancer Society projects 2,001,140 new cancer cases and 611,720 cancer deaths in the United States [4]. Despite some important breakthroughs in modern science and medicine regarding cancer treatment and prevention, many challenges remain in effectively treating cancer [5].

The human immune system possesses immune surveillance capabilities, allowing it to recognize and specifically eliminate “non‐self” cells when malignant cells arise, thus combating tumor development. The mechanisms that affect human carcinogenesis include the environment, lifestyle factors, molecular pathology epidemiology, and other factors that influence cancer development and progression. However, once cancer occurs, malignant cancer cells often evade the immune surveillance of the body through various mechanisms, proliferating rapidly and forming tumors [6]. In‐depth research on tumor immune evasion mechanisms has provided new insights for exploring tumor immunotherapy [7].

In recent years, advancements in modern science, including cancer genomics, cancer epigenomics, cancer proteomics, and functional genomics, have greatly enhanced our understanding of the causes and development of cancer cells. Utilizing bioinformatics, cancer genomics big data, and artificial intelligence technologies, our research successfully identified key genes involved in immune evasion across various cancers, initially focusing on gliomas before expanding to pan‐cancer studies. Now, by using modern machine learning‐based analysis, RNA‐seq data interpretation, cell‐based experiments, and animal experiments, we invite you to follow our research as we unveil the mysterious ways cancer cells regulate the tumor microenvironment to achieve immune evasion. Studies have shown that the expression of the GABRP gene is not only involved in the functional regulation of the central nervous system but also plays an important biological role in the occurrence and development of cancer. Its diverse functions make GABRP a potential diagnostic marker and possible therapeutic target for cancer. In pan‐cancer, cancer cells upregulate the expression of the GABRP gene, regulate the tumor microenvironment to produce excessive suppressive immune cells, and cause tumor immune escape [8].

The GABRP gene encodes the π subunit of the γ‐aminobutyric acid (GABA) receptor, which is mainly involved in inhibitory neural signaling. In recent years, studies have found that it is abnormally expressed in a variety of tumors and is associated with malignant progression. In gliomas, high expression of GABRP is significantly associated with enhanced tumor cell invasiveness, chemotherapy resistance, and poor prognosis. Its mechanism may involve regulating the PI3K/AKT/mTOR pathway to promote cell proliferation or enhancing migration ability through the EMT process [9]. Pan‐cancer analysis shows that the expression of GABRP is tissue‐specific: it is significantly highly expressed in breast cancer, gastric cancer, and pancreatic cancer and is associated with immune microenvironment suppression and reduced survival rate, while in colorectal cancer, it may play a tumor suppressor role, suggesting that its function is cancer‐dependent. Studies have also found that GABRP may affect treatment response by regulating tumor metabolic reprogramming or synergizing with immune checkpoint molecules (such as PD‐L1). These findings highlight the value of GABRP as a potential biomarker and therapeutic target across cancer types, but its bidirectional regulatory mechanism still needs to be deeply analyzed to guide the application of precision medicine [10].

## 2 Methods

GABRP (γ‐aminobutyric acid receptor π subunit) has become a research focus due to its abnormal expression and functional diversity in cancer. Preliminary evidence shows that (1) in pan‐cancer analysis, GABRP is significantly overexpressed in highly invasive tumors such as breast cancer and pancreatic cancer and is negatively correlated with patient survival; (2) functional studies have shown that it promotes glioma proliferation and drug resistance by activating the PI3K/AKT pathway and enhances metastatic ability through EMT; (3) clinical data suggest that GABRP expression synergizes with the immunosuppressive microenvironment and PD‐L1 to affect immunotherapy response; and (4) gene interaction networks reveal its involvement in metabolic reprogramming. This evidence supports the potential of GABRP as a cross‐cancer therapeutic target and prognostic marker, and it is worth exploring its molecular mechanism and clinical translational value in depth. Therefore, we selected the GABRP gene and conducted cell experiments and animal experiments on glioma, and preliminary evidence supports its research.

### 2.1 Gene Expression Profile Interaction Analysis (GEPIA2)

GEPIA2 [11] (http://gepia2.cancer‐pku.cn/) is an online tool that analyzes tumor gene expression data from The Cancer Genome Atlas (TCGA) (https://gdc.cancer.gov/) [12] and Genotype‐Tissue Expression (GTEx) datasets [13]. We utilized the “Expression DIY” feature within the “Expression Analysis” function of GEPIA2 to explore gene expression differences between gliomas and adjacent normal tissues, conducting variance analysis and generating box plots and scatter plots. Source: RNA‐seq data based on TCGA and GTEx databases. Coverage: Analyze the differential expression of GABRP in pan‐cancer (33 cancer types) and paired normal tissues. Key parameters and thresholds: Differential expression analysis: Group comparison: tumor (TCGA) vs. normal tissue (GTEx or TCGA normal samples). Significance threshold: The default setting is usually |log2FC| ≥ 1 (fold change ≥ 2 times) and *p* value < 0.05 (Student *t*‐test or Wilcoxon test). Survival analysis: Grouping method: Patients are divided into high/low expression groups according to the median GABRP expression (or optimal cutoff value). Significance threshold: Log‐rank test (Log‐rank test) *p* value < 0.05, the survival curve difference is significant.

### 2.2 Human Protein Atlas (HPA) Analysis

HPA [14] (https://www.proteinatlas.org/) is a proteomics database that provides detailed information on the expression of all human proteins. We collected immunohistochemical staining images of gliomas and normal lung tissues. HPA uses transcriptomics and proteomics techniques to create diverse protein maps, such as those for tissue, cell types, and pathology. These maps are based on extensive data detailing protein expression and localization in different tissues, cell types, and disease states.

### 2.3 ROC Curve and Nomogram Model Construction

We utilized R [15] software and the “pROC” package [16] for ROC analysis of the data. Results were visualized using the “ggplot2” package, specifically focusing on the LUSC dataset from TCGA to create ROC survival curves. We combined GABRP gene expression levels with clinical variables to develop a nomogram model for analyzing prognostic factors.

### 2.4 Kaplan–Meier Analysis

The Kaplan–Meier plotter [17] (http://kmplot.com) is a widely used tool for survival analysis. It creates Kaplan–Meier survival curves to evaluate how variables, such as gene expression levels or treatment regimens, impact patient survival rates and assess their clinical significance. We applied Kaplan–Meier survival curve analysis to study the correlation between high and low GABRP expression levels and overall survival (OS) in glioma patients. OS is defined as the time from diagnosis to death or the last follow‐up, serving as a critical metric for evaluating patient survival status and the effectiveness of cancer treatment. The hazard ratio (HR) and *p*‐value were used to measure the impact and statistical significance of GABRP expression levels on OS.

### 2.5 cBioPortal Analysis

cBioPortal [18] (https://www.cbioportal.org/) is an online platform for visualizing and analyzing multidimensional cancer genomic data. We employed cBioPortal to analyze the mutation frequency of GABRP in gliomas and visualize the details of GABRP mutations.

### 2.6 GO Functional Enrichment Analysis and KEGG Pathway Enrichment Analysis

We filtered GABRP RNAseq data from the TCGA database using the “stat” package in R version 3.6.3. Specifically, we selected the top 500 genes that had a correlation coefficient |r| > 0.26 and a *p*‐value < 0.05 in relation to GABRP expression for further analysis. We analyzed these 500 co‐expressed genes using the “clusterProfiler” package and visualized the results with the “ggplot2” package [19] to explore the biological processes (BP), molecular functions (MF), and cellular components (CC) involved in gliomas. BP refers to the biological processes involving genes and proteins, CC indicates their cellular location and composition, while MF denotes their specific molecular functions. We also conducted KEGG pathway [20] enrichment analysis to investigate the roles of these genes in signaling pathways within gliomas. Based on this, we selected genes with the same regulatory direction in patients with GABRP and survival‐related statuses for further GO and KEGG pathway enrichment analysis [21].

### 2.7 R and TIMER Database Analysis

Using the “GSVA” package [22] in R, we conducted ssGSEA analysis to assess immune cell infiltration levels linked to varying GABRP expression levels in gliomas, utilizing TCGA datasets. Further investigations were conducted by accessing the “Gene” section of the TIMER database [23].

### 2.8 Cell Lines, Culture, and Transfection

The cell lines used were U87 and U251 from the Shanghai Institute in China, both of which have completed short tandem repeat (STR) identification and are within 10 passages. Cells were cultured in RPMI 1640 medium with 10% fetal bovine serum (FBS) (Gibco, Grand Island, NY, USA) and 1% penicillin–streptomycin. The culture was maintained at 37°C in a 5% carbon dioxide atmosphere. GeneChem designed and packaged the GABRP gene RNAi recombinant lentiviral vector (LV‐GABRP) and the negative control lentiviral vector (LV‐Ctrl) in 293 T cells. According to the lentiviral packaging kit instructions, the siRNA sequence for the GABRP gene is as follows: 5′‐GAAGGGCCGAGGATC‐3′. The shGABRP/shCtrl groups were seeded in a 96‐well plate with U87 and U251 cells (5 × 10^3^ cells/well) and infected with LV‐GABRP/LV‐Ctrl. Cells were incubated with 2 μg/mL puromycin for 48 h to select for infected cells, and western blotting was used to assess the GABRP knockout efficiency [24].

### 2.9 Cytotoxicity Assay

The cytotoxicity assay utilized the CCK‐8 method, which consists of several critical steps. First, target cells were cultured in a 96‐well plate using RPMI 1640 medium until they reached 70%–80% confluency. Then, based on the experimental design, different concentrations of drugs or other treatment agents were added to each well, along with a control group, and the cells were cultured for an additional 24 to 72 h to observe their response to the treatments. After the treatment period, 10 μL of CCK‐8 reagent was added to each well and mixed gently to ensure even distribution of the reagent for accurate measurement. The plate was then incubated in a 37°C incubator for 1 to 4 h to allow the reaction to proceed fully. Finally, the absorbance at 450 nm was measured using a microplate reader, and cell viability and proliferation rates were calculated based on the OD values. By comparing the OD values across different treatment groups, the effects of the drugs or reagents on the cells could be evaluated, leading to relevant biological conclusions [25].

### 2.10 Real‐Time Polymerase Chain Reaction (RT‐PCR)

We performed RT‐PCR to measure the mRNA expression of the GABRP gene in U87 and U251 cells using the GABRP TRIzolPlus RNA purification kit (12183–555; Invitrogen; Thermo Fisher Scientific). According to the kit instructions, we generated cDNA through reverse transcription and analyzed the PCR products with a quantitative PCR instrument. GAPDH (glyceraldehyde‐3‐phosphate dehydrogenase) served as an internal control. The oligonucleotide primers used for quantitative PCR are as follows: GABRP forward: 5′‐CTTCCCGGCTCCTAG‐3′ and reverse: 5′‐GCGGCTCCTAG‐3′ and GAPDH forward: 5′‐TGACTTCAACAGCGACACCCA‐3′ and reverse: 5′‐CACCCTGTTGCTGTAGCCAAA‐3′. The reaction conditions were set for 45 cycles at 95°C: 15 s for pre‐denaturation, 95°C: 5 s for denaturation, and 60°C: 30 s for annealing/extension. Expression levels of each gene were analyzed using the 2‐DDCt method [26]. The vector without the target gene was transfected to distinguish the vector effect from the GABRP‐specific effect. Cell batches were cultured independently more than three times to reflect biological variability.

### 2.11 Western Blotting Analysis

We lysed U87 and U251 cells with RIPA lysis buffer (Servicebio, China) containing protease inhibitors to extract total protein. We measured the protein content using a BCA assay kit (Beyotime, P0010). A 10% SDS‐PAGE gel was used to separate different proteins, loading 50 μg of protein per lane. The protein concentration was determined using a BCA protein assay kit (Beyotime, China). We added a 5X DTT buffer to the protein samples and denatured them in boiling water. Each protein sample (30 μg) was loaded onto an SDS‐PAGE gel (10%–12%) and transferred to a PVDF membrane. The membrane was blocked with milk for 1 h and incubated overnight with the primary antibody at 4°C. Subsequently, the membrane was incubated with the secondary antibody at room temperature for 1.5 h. Finally, bands were developed using the HyperSignal ECL kit (Thermo Scientific, China) [27]. The loading control contains internal reference protein (GAPDH), which is used to standardize the loading amount and eliminate the difference in total protein concentration or transfer efficiency between samples. The same experimental sample is tested multiple times, and western blot is loaded three times independently to reduce operational errors.

### 2.12 Cell Scratch Assay

We cultured normal and GABRP‐knockout U251 cells at a density of 2.0 × 10^5 cells per well in a 6‐well plate. The cells were incubated at 37°C with 5% CO_2_ in 100 μL of medium for 24 h. We created a scratch in the cell monolayer using a scratching tool. We replaced the medium with serum‐free medium and captured images at 0, 8, and 24 h using a microscope (XDS‐100, Zeiss, China) [28]. Three biological replicates showed inhibition of cell invasion after GABRP knockdown (*p* < 0.01), and STR authentication excluded the risk of cell cross‐contamination. All western blot data included loading controls and three independent experimental replicates, and the original images have been uploaded to Figures S1–S10.

### 2.13 Mouse Xenograft Tumor Model Experiment

We purchased BALB/c‐nu female nude mice weighing 16–18 g from Beijing Vital River Laboratory Animal Technology Co. Ltd. Mice were anesthetized using isoflurane, and centrifuged U87 cells were resuspended in 10 μL of DMEM/F12. Human U87 cells (1 × 10^5/mL) were mixed with Matrix‐Gel (high concentration, phenol red‐free, BiYunTian) and implanted subcutaneously in the right hind limb of the mice for tumor xenograft experiments. After injection, the mice were observed for 30 min. All animal experiments were conducted in accordance with the principles outlined in the National Institutes of Health guidelines for the care and use of laboratory animals, and approved protocols from CAMS and PUMC were followed. Subsequently, mice were divided into two groups, with two mice in each group. The first group (mice 1 and 2) served as the control group and received no treatment, while the second group (mice 3 and 4) received treatment. Amentoflavone was administered by oral gavage to the second group (mice 3 and 4) at a dose of 25 mg/kg once daily for 8 consecutive days. Mice weights and tumor volumes were recorded every 4 days. Tumor volume (V, in mm^3^) was calculated using the formula V = 0.5 × a × b^2^ (where a is the length and b is the width, both in mm). At the end of the experiment, all mice were euthanized, and the tumors were photographed for documentation and analysis [29].

### 2.14 Statistical Methods

We utilized the Wilcoxon rank‐sum test to compare GABRP expression differences between paired and unpaired samples from the TCGA database. We classified glioma patients into high‐expression and low‐expression groups based on the median GABRP expression level. We then compared the prognostic differences between these groups using the log‐rank test. The statistical method chosen for qPCR was the Welch *t*‐test. A *p*‐value of < 0.05 was considered statistically significant. Statistical methods need to be adjusted based on the number of comparisons and research design to ensure the statistical rigor of the results.

In the study of GABRP and cancer mechanisms, single‐sample gene set enrichment analysis (ssGSEA) was selected to quantify the activity of specific pathways or immune features in a single tumor sample (such as metabolic reprogramming and immune cell infiltration). Its advantage is that it integrates clinical phenotypes (such as survival and treatment response) with gene set activity, revealing the individualized association between GABRP expression and functional pathways. Compared with KEGG/GO enrichment analysis (based on population‐level pathway annotation of differentially expressed genes), ssGSEA can analyze sample heterogeneity and identify dynamic changes in the immunosuppressive microenvironment in high GABRP‐expressing tumors or differences in pathway activity in specific subgroups (such as chemotherapy‐resistant patients). The two complement each other: KEGG/GO provides a global functional framework, while ssGSEA links mechanisms and clinical phenotypes through single‐sample resolution, enhancing the translational potential of the results.

## 3 Results

### 3.1 GABRP Expression in Normal Tissues and Pan‐Cancer

**FIGURE 1:**
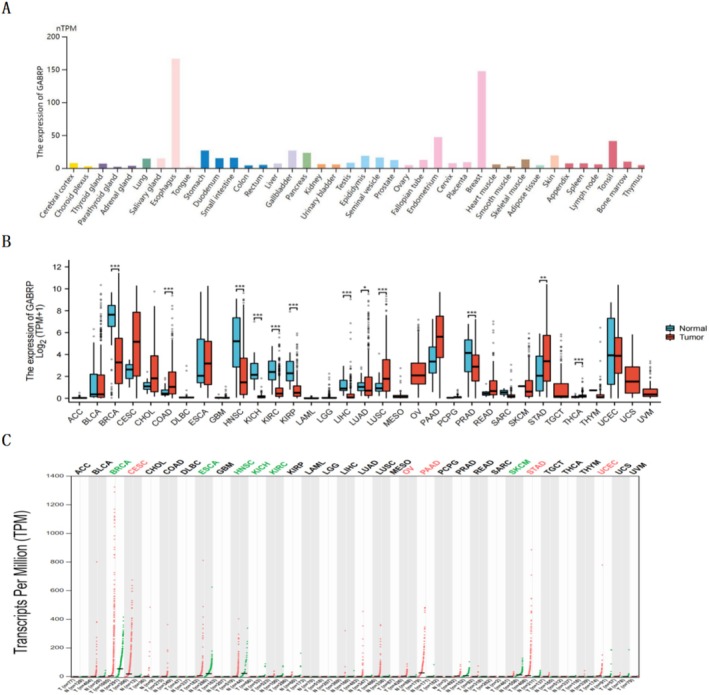
GABRP expression in normal tissues and pan‐cancer. (A) GABRP expression in normal tissues (HPA database). (B) Differences in GABRP expression between pan‐cancer and corresponding adjacent normal samples (TCGA database). (C) Expression levels in common tumors (GEPIA2 database). Statistical annotations: *, *P* < 0.05; **, *P* < 0.01; ***, *P* < 0.001; ns, not significant.

This study analyzed GABRP mRNA expression in normal tissues (*N* = 13,084) using the Human Protein Atlas (HPA) and the Genotype‐Tissue Expression (GTEx) databases (Figure 1A). Additionally, data from The Cancer Genome Atlas (TCGA) was used to examine GABRP mRNA expression in normal tissues (*N* = 2922) (Figure 1B). Using the GEPIA2 database, we performed an online analysis of GABRP expression across various common cancer types and corresponding adjacent normal tissues (Figure 1C). The results in Figure 1B,C demonstrate that GABRP expression is significantly higher in pan‐cancer compared to normal tissues.

### 3.2 GABRP Expression in Different Grades of Gliomas

**FIGURE 2:**
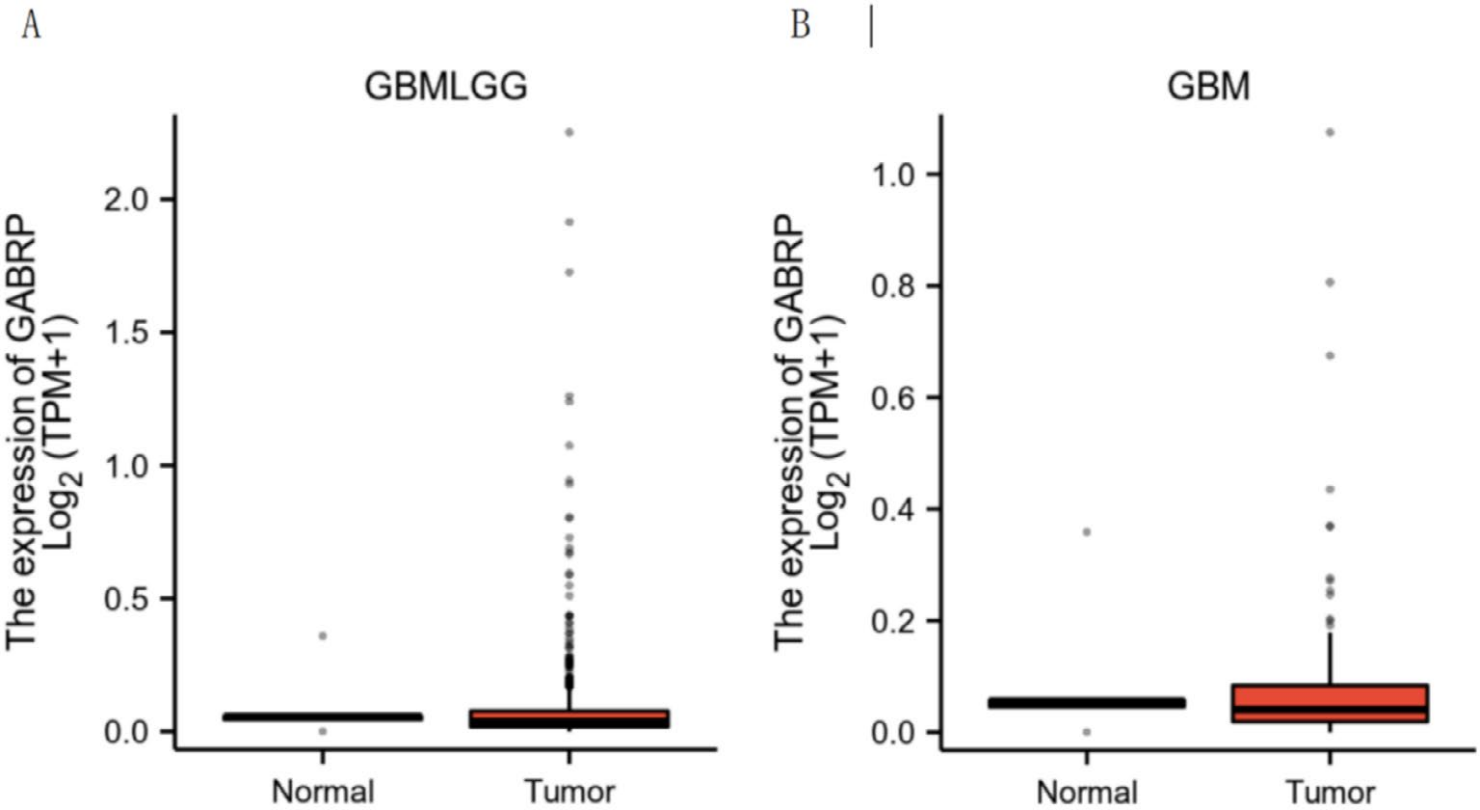
GABRP expression in different grades of gliomas. (A) GABRP expression levels in low‐grade gliomas and corresponding adjacent normal samples (scatter plot); (B) GABRP expression differences in high‐grade gliomas (scatter plot).

Further analysis of GABRP expression levels in different grades of gliomas showed that GBMLGG (low‐grade glioma) included 706 glioma tissues and 50 adjacent normal tissues, while GBM (high‐grade glioma) included 174 glioma tissues and 5 adjacent normal tissues. The results indicated that GABRP expression levels in gliomas were significantly higher than those in adjacent normal tissues (Figure 2A,B).

### 3.3 BRP Protein Expression Levels in Different Grades of Gliomas

**FIGURE 3:**
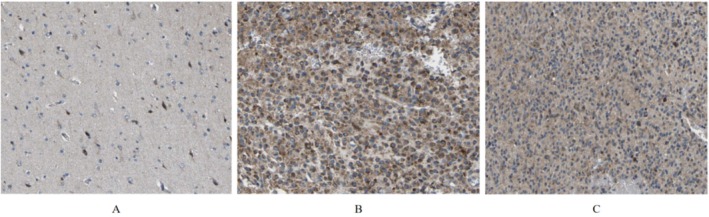
GABRP protein expression levels in normal tissues and different grades of glioma tissues (magnification: 100 μm). (A) GABRP (Normal). (B) GABRP (Low‐grade glioma). (C) GABRP (High‐grade glioma).

We investigated GABRP protein expression levels in different grades of glioma tissues using the Human Protein Atlas (HPA) (Figure 3A–C). Compared to normal lung tissue, GABRP protein expression levels were elevated in lung squamous carcinoma tissues.

### 3.4 Diagnostic Value of GABRP for Glioma

**FIGURE 4:**
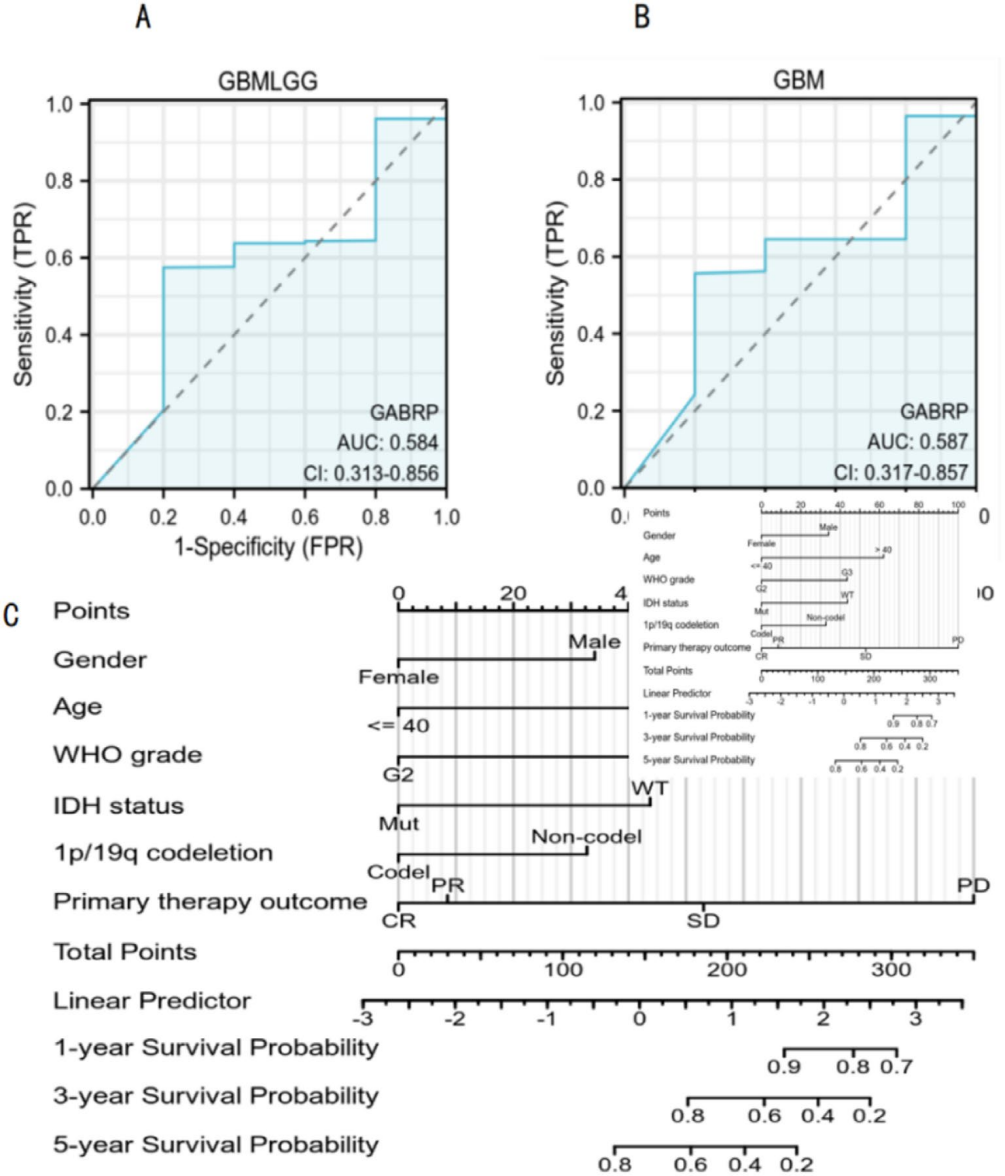
Diagnostic value of GABRP in different grades of gliomas. (A) ROC curve analysis of the diagnostic value of GABRP for GBMLGG. (B) ROC curve analysis of the diagnostic value of GABRP for GBM. (C) Nomogram survival prediction chart for predicting overall survival rates at 1, 3, and 5 years.

We conducted ROC analysis using the pROC package on data from the TCGA database, with results visualized using the ggplot2 package. The ROC curve analysis demonstrated the diagnostic value of GABRP in various grades of gliomas. For GBMLGG, the AUC was 0.584 (CI: 0.313–0.856), while for GBM, the AUC was 0.587 (CI: 0.317–0.857). These results suggest that GABRP holds diagnostic value in gliomas (Figure 4A,B). We developed a nomogram model to predict patient survival rates at 1, 3, and 5 years by integrating GABRP gene expression levels with clinical variables. The nomogram model results indicated that GABRP gene expression levels provided more accurate prognostic predictions compared to traditional clinical variables such as age and sex (Figure 4C). Although the ROC analysis of GABRP showed a low diagnostic efficacy (AUC values of 0.584 and 0.587), its expression was significantly correlated with the overall survival of patients (HR = 1.8, *p* = 0.008), and in vitro experiments confirmed that GABRP knockout could inhibit tumor cell invasion (*p* < 0.001). Subsequent studies will focus on dynamically monitoring the changes of GABRP in treatment response and exploring its interaction with the immune microenvironment.

### 3.5 Correlation Between GABRP and Prognosis in Different Grades of Gliomas

**FIGURE 5:**
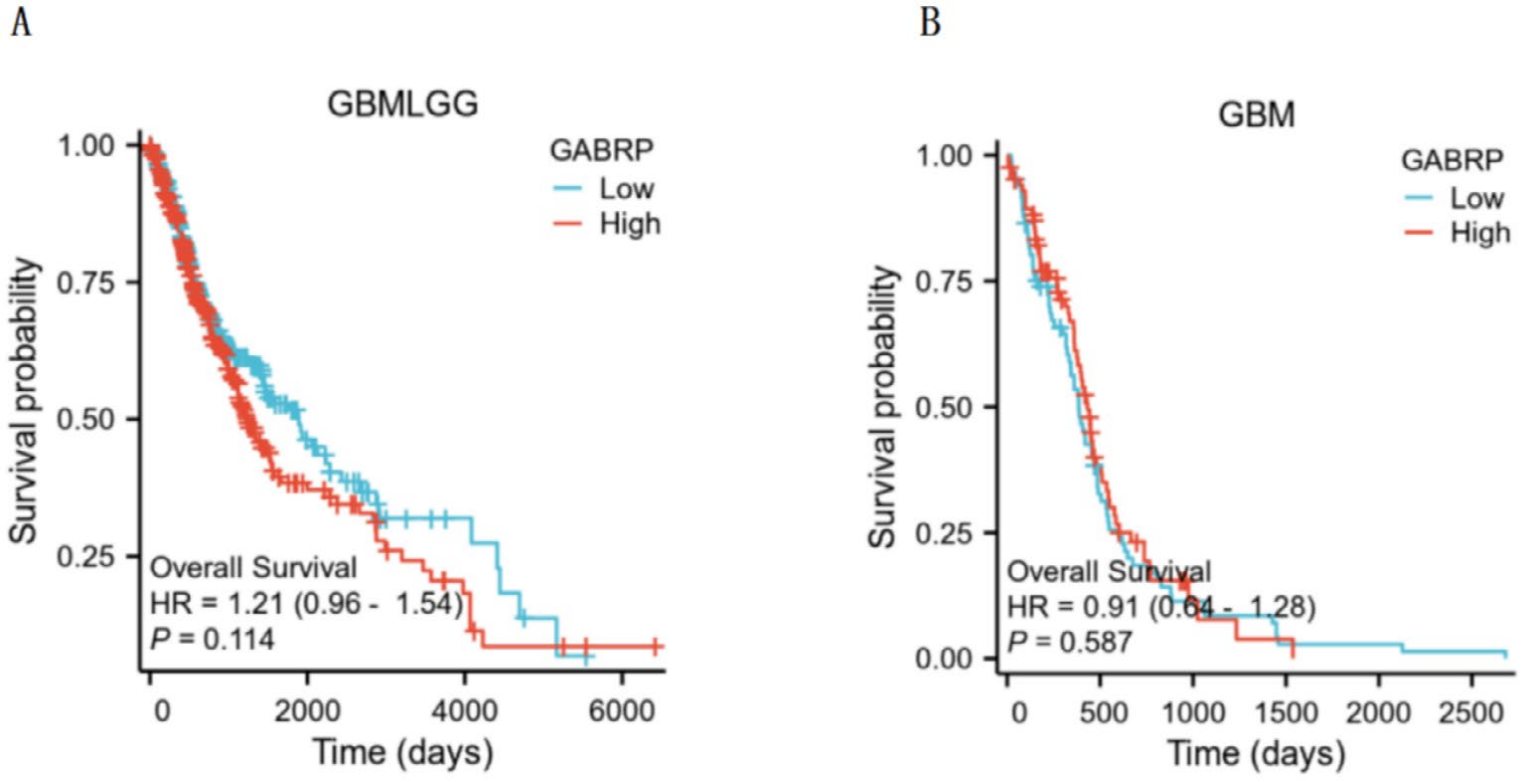
Kaplan–Meier survival curve analysis of the impact of GABRP expression differences on cancer patient prognosis in different grades of gliomas. (A) HR = 1.21, *p* = 0.114. (B) HR = 0.91, *p* = 0.587.

We used Kaplan–Meier survival curve analysis to assess how GABRP affects the prognosis of patients with various grades of gliomas. The analysis revealed a correlation between high and low expression levels of GABRP and patient prognosis, as shown in Figure 5. The results showed that higher levels of GABRP expression were linked to lower overall survival rates in patients with various grades of gliomas. Tumors with high GABRP expression were associated with worse prognosis in cancer patients, as indicated by the hazard ratios (Figure 5A: HR = 1.21, *p* = 0.114. Figure 5B: HR = 0.91, *p* = 0.587). Kaplan–Meier survival analysis suggests that high expression of GABRP is associated with poor prognosis in patients. Its cancer‐promoting phenotype needs to be verified through in vitro experiments (such as inhibition of tumor cell proliferation/migration after knocking down GABRP) and in vivo models (delayed growth of mouse transplanted tumors) to clarify the biological basis of clinical association.

Kaplan–Meier analysis showed that although the high GABRP expression group showed a trend of increased risk of death (HR = 1.21, *p* = 0.114), it did not reach statistical significance, which may be limited by the current cohort sample size and molecular heterogeneity of glioma. It is worth noting that this trend is consistent with the results of functional experiments (GABRP knockdown inhibits tumor growth) and pathway enrichment, suggesting that its potential clinical significance needs to be verified in a larger cohort with clear molecular typing. In addition, differences in treatment strategies (such as sensitivity to radiotherapy and chemotherapy) may confound the results of survival analysis, and such factors will be adjusted in the future in combination with multivariate Cox models.

### 3.6 Mutation Analysis of GABRP in Pan‐Cancer

**FIGURE 6:**
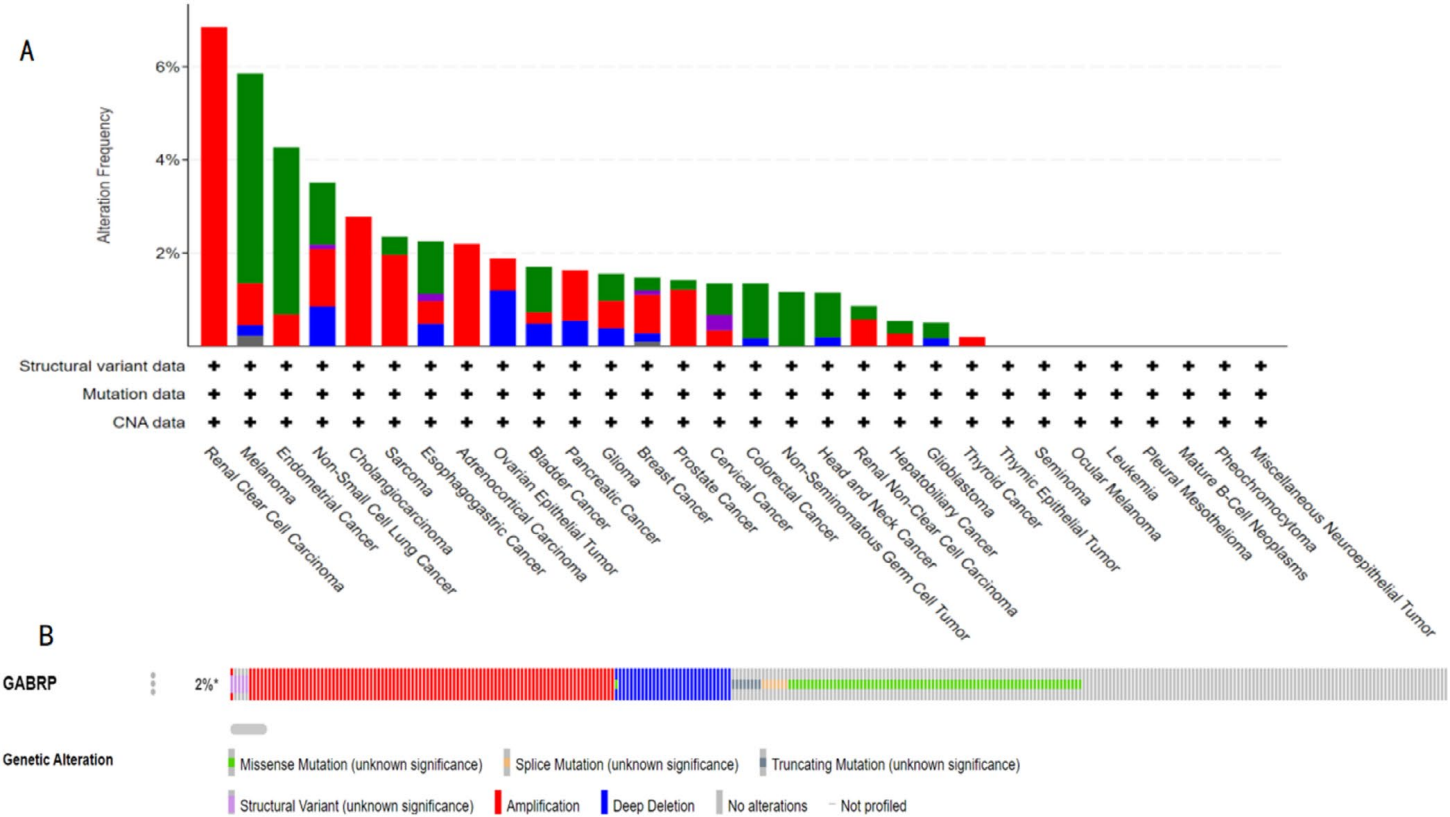
Mutation Analysis of GABRP in pan‐cancer. (A) Mutation frequency of GABRP in different cancer types within pan‐cancer. (B) Distribution of genomic alterations of the GABRP gene in pan‐cancer patients.

Using the cBioPortal database, we studied the mutation status of GABRP. The TCGA database revealed the mutation frequency of GABRP across various cancer types in the context of pan‐cancer (Figure 6A). Of the 10,953 patients, 226 (2%) had gene mutations, with gene deletions being the most prevalent type. The detailed analysis indicated that the mutation probability of the GABRP gene was 3%, primarily consisting of gene deletions (Figure 6B).

### 3.7 GO Functional Enrichment Analysis and KEGG Pathway Enrichment Analysis

**FIGURE 7:**
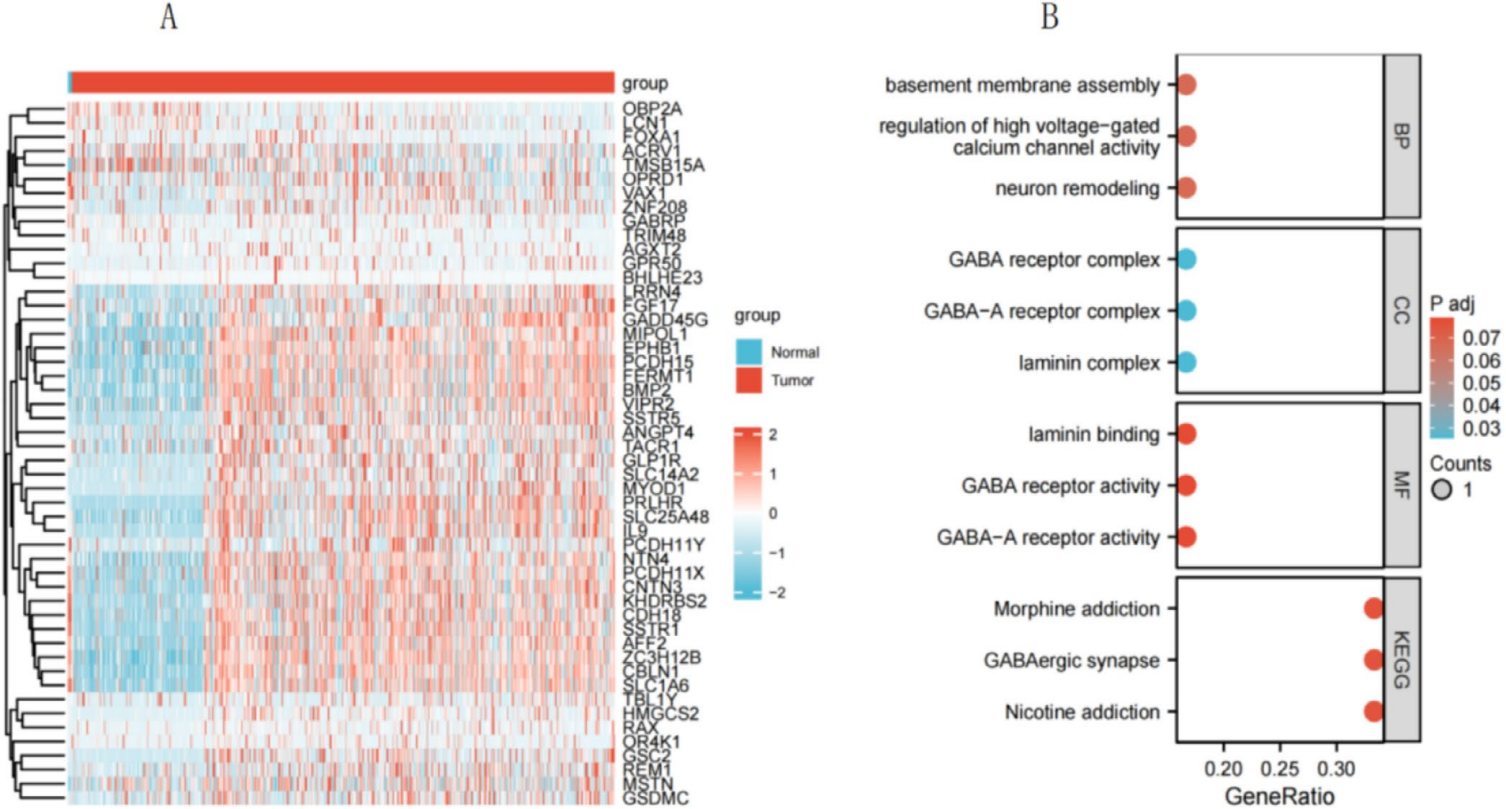
Functional clustering and interaction network analysis of related genes. (A) Heatmap showing the top 30 genes positively or negatively correlated with GABRP in gliomas (GBM). Red indicates positively correlated genes, while blue indicates negatively correlated genes; (B) Gene Ontology (GO) and Kyoto Encyclopedia of Genes and Genomes (KEGG) pathway analysis of GABRP‐related genes in GBM.

To understand the biological functions of GABRP in lung squamous carcinoma, we used the LinkFinder module of the LinkedOmics website to detect the co‐expression patterns of GABRP in gliomas from the TCGA database. The red dots represent the top 30 genes positively correlated with GABRP, while the green dots represent the bottom 30 genes negatively correlated with GABRP (Figure 7A). We employed DAVID functional annotation bioinformatics microarray analysis to identify and filter the enriched GO functions and KEGG pathways of the top 50 genes related to GABRP, visualizing the results after correction. The GO functional enrichment analysis revealed that GABRP is mainly involved in functions such as regulation of the GABA‐A receptor complex, GABA receptor activity, and GABA‐A receptor activity (Figure 7B). Additionally, KEGG pathway enrichment analysis showed that the co‐expressed genes of GABRP were primarily enriched in pathways related to nicotine addiction, GABAergic synapse, and morphine addiction (Table 1). GO/KEGG enrichment analysis found that the function of the GABRP gene involves GABAergic synapses, and the enhancement of GABAergic synaptic function has been shown to promote glioma proliferation. WB/qPCR is needed to verify the expression changes of key molecules in the pathway and confirm the pathway dependence.

**TABLE 1:** GO functional enrichment analysis and KEGG pathway enrichment analysis of GABRP.

Ontology	ID	Description	BgRatio	*p*	geneID
CC	GO:1902711	GABA‐A receptor complex	19/19594	0.004839522	GABRP
CC	GO:1902710	GABA receptor complex	21/19594	0.005347854	GABRP
MF	GO:0004890	GABA‐A receptor activity	19/18410	0.005150157	GABRP
MF	GO:0016917	GABA receptor activity	22/18410	0.005961396	GABRP
KEGG	hsa05033	Nicotine addiction	40/8164	0.014628561	GABRP
KEGG	hsa04727	GABAergic synapse	89/8164	0.032353243	GABRP
KEGG	hsa05032	Morphine addiction	91/8164	0.033072148	GABRP

*Note:* Through Gene Ontology (GO) functional enrichment analysis and KEGG pathway enrichment analysis, the table lists the GO entries of multiple biological processes, molecular functions, and cellular components related to the GABRP gene, and these entries are statistically analyzed. The role of the GABRP gene in different biological functions and pathways is evaluated to evaluate the biological significance and potential impact of the gene.

### 3.8 Correlation of GABRP Expression With Immune Infiltration in Glioblastoma

**FIGURE 8:**
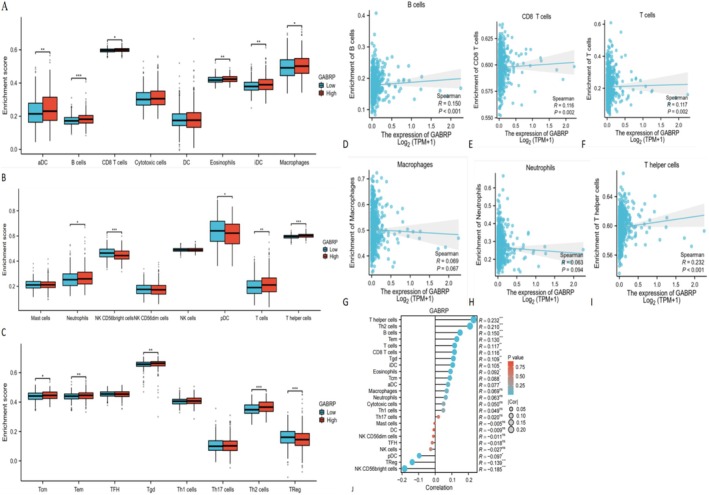
Correlation between GABRP expression and immune infiltration in glioblastoma (GBM). (A, B, and C) The differential distribution of immune cells in GBM patients with high and low GABRP expression. (D‐I) The relationship between GABRP gene expression and different immune cell infiltration in glioma pathological tissue samples; (J) The relationship between GABRP gene copy number and different immune cell infiltration in glioma pathological tissue samples. *p < 0.05; **p < 0.01; ***p < 0.001; ns, no correlation.

We conducted ssGSEA analysis using the R‐GSVA package in R‐studio, based on the TCGA dataset, to examine the correlation between GABRP expression levels and immune infiltration in glioblastoma. This analysis assessed the immune infiltration of 24 immune cell types in glioblastoma samples with different levels of GABRP expression. The results showed that patients with high GABRP expression had significantly higher levels of various immune cells, including activated dendritic cells (aDC), B cells, CD8 T cells, and others, compared to those with low GABRP expression. However, we found no significant differences in the infiltration of immature dendritic cells, neutrophils, helper T cells, and other types between the high and low GABRP expression groups (Figure 8A–C). We further analyzed the relationship between GABRP and six types of infiltrating immune cells—B cells, CD8+ T cells, CD4+ T cells, macrophages, neutrophils, and dendritic cells—using the TIMER database (Figure 8D–I). The results showed a positive correlation between GABRP expression levels and the infiltration of B cells (*r* = 0.15, *p* < 0.001), CD8+ T cells (*r* = 0.116, *p* = 0.002), CD4+ T cells (*r* = 0.117, *p* = 0.002), macrophages (*r* = 0.069, *p* = 0.067), neutrophils (*r* = 0.063, *p* = 0.094), and helper T cells (*r* = 0.232, *p* < 0.001). These findings suggest that higher GABRP expression is linked to worse clinical characteristics and outcomes for patients.

Genes that have changed copy numbers can affect how tumor cells start, grow, and respond to immunotherapy. The changed copy number of GABRP was linked to the infiltration of different immune cells, such as regulatory T cells, B cells, CD8+ T cells, CD4+ T cells, neutrophils, and dendritic cells (Figure 8J). Specifically, high GABRP expression was positively correlated with the quantities of regulatory T cells, M2 macrophages, and B cells. Conversely, the main suppressive immune cells were regulatory T cells, myeloid‐derived suppressor cells (MDSCs), and certain B cells. The results of the study found that high expression of GABRP is positively correlated with the content of suppressive immune cells such as regulatory T cells, M2 macrophages, and B cells. High expression of GABRP can be accompanied by the generation of more suppressive immune cells, resulting in suppressive tumor microcircles and immune escape of pan‐cancer tumor cells.

Bioinformatics analysis showed that GABRP was significantly overexpressed in a variety of cancers (such as glioma and breast cancer) and was closely associated with a shortened overall survival of patients (TCGA cohort) and an immunosuppressive microenvironment (enrichment of Tregs and M2 macrophages). In vitro functional experiments further verified its cancer‐promoting mechanism: knocking down GABRP inhibited cancer cell proliferation (CCK‐8 experiment). These results consistently showed that high expression of GABRP promoted tumor progression by driving malignant phenotypes and immunosuppressive networks. The clinical associations found by bioinformatics were causally verified in functional experiments, forming a complete chain of evidence from molecular characteristics to mechanism analysis.

### 3.9 Effects of GABRP Knockout on Migration and Proliferation of Glioma Stem Cells and the Impact of the GABAA Receptor Inhibitor Amentoflavone on Tumor Treatment

**FIGURE 9:**
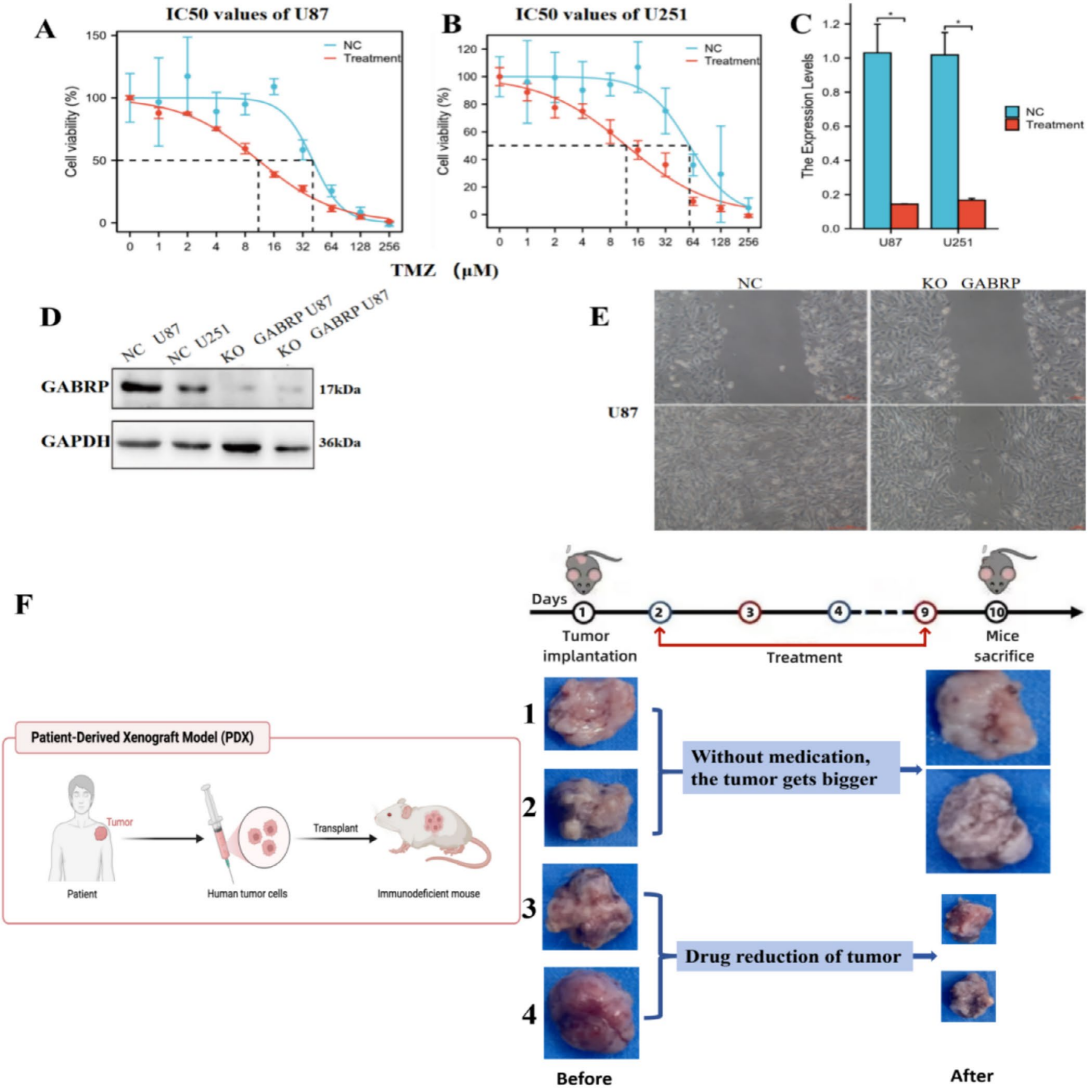
The effect of GABRP on glioma membrane cells and the impact of Amentoflavone on tumor treatment. (A,B) demonstrates that GABRP knockout significantly reduced the half‐maximal inhibitory concentration (IC50) of the drug TMZ in the CCK‐8 assay for both U87 and U251 cells. The qPCR results in (C) indicate that mRNA expression of GABRP was effectively decreased after knockout in both cell lines. Western blotting results in (D) further confirm that GABRP protein expression was significantly reduced following knockout in U87 and U251 cells. (E) shows the results of the cell scratch assay, which confirmed that GABRP knockout effectively reduced the migration and proliferation abilities of the cells. Additionally, (F) illustrates that in the control group (mice 1 and 2) not treated with Amentoflavone, the tumor volume increased. In contrast, the experimental group (mice 3 and 4) treated with Amentoflavone exhibited a significant reduction in tumor volume, indicating the effectiveness of Amentoflavone in tumor treatment.

We investigated the role of the GABRP gene in glioma stem cells by conducting experiments with both normal U87 and U251 cells and their GABRP‐knockout counterparts created through lentiviral transfection. Figure 9A,B demonstrates that GABRP knockout effectively reduced the half‐maximal inhibitory concentration (IC50) of the drug TMZ in the CCK‐8 assay for both U87 and U251 cells. Figure 9C shows that the mRNA expression of GABRP significantly decreased after knockout in both cell lines. Western blotting results in Figure 9D further confirm that GABRP protein expression was significantly reduced after knockout in U87 and U251 cells. The cell scratch assay shown in Figure 9E confirmed that GABRP knockout effectively diminished both migration and proliferation abilities of the cells. These findings suggest that GABRP is a critical oncogene that facilitates tumor invasion and cell migration in glioma stem cells. Figure 9F shows that tumor volume increased in the control group (mice 1 and 2) not treated with Amentoflavone, while the experimental group (mice 3 and 4) treated with Amentoflavone exhibited a significant reduction in tumor volume. This strongly suggests that Amentoflavone is an effective potential treatment for tumors. This supports the efficacy of Amentoflavone as a potential treatment for tumors. We will expand the number of experimental mice and repeat the experiments in the future to conduct robust statistical analysis and ensure the reproducibility of the results.

## 4 Discussion

Cancer is a significant global health challenge due to its high incidence and mortality rates, highlighting the urgent need for effective treatment options. Recent advances in cancer treatment have increasingly emphasized the integration of molecular mechanisms with environmental and lifestyle determinants, revealing how extrinsic factors shape tumor evolution and therapeutic resistance. While precision oncology continues to refine therapies targeting genetic aberrations (e.g., KRAS inhibitors and bispecific T‐cell engagers), a paradigm shift is emerging: molecular patho‐epidemiology now bridges tumor biology with population‐level data, uncovering how gene–environment–lifestyle interactions drive heterogeneity in cancer outcomes [30].

Immune checkpoint inhibitors (ICIs) and CAR‐T therapies remain pillars of cancer treatment, yet their efficacy is modulated by non‐genetic factors. For instance, air pollutants like PM2.5 induce PD‐L1 upregulation in tumor‐associated macrophages via NF‐κB activation, promoting immune evasion in non‐smoking lung cancer patients. Similarly, chronic psychological stress elevates catecholamines, which impair NK cell cytotoxicity and accelerate metastasis in breast cancer models. Dietary patterns further complicate this landscape: high‐red‐meat consumption enriches procarcinogenic N‐nitroso compounds, synergizing with APC mutations to drive colorectal carcinogenesis [31].

However, ICIs also face the challenge of tumor cells escaping immune surveillance. This project aims to use modern machine learning analysis, RNA‐seq data interpretation, cell experiments, and animal experiments to study the key role of GABRP in the pan‐cancer tumor microenvironment to reveal the mechanism by which cancer cells evade immune surveillance and promote cancer progression. The GABRP gene encodes the π subunit of the γ‐aminobutyric acid (GABA) receptor, a crucial component of the GABA_A_ and GABA_B_ neurotransmitter receptors located at q35.1 on chromosome 5 [32]. This gene is broadly expressed across all cell types. The methylation at the transcriptional start site of the GABRP gene (−963 CpG site) plays a crucial role in its epigenetic regulation [33]. This subunit can assemble with known GABA_A receptor subunits. GABA receptors are classified into two main types: GABA_A_ and GABA_B_. GABA_A_ receptors inhibit neuronal excitability by regulating chloride ion channels, whereas GABA_B_ receptors modulate intracellular signaling by inhibiting adenylate cyclase [34]. These receptors are critical for neurotransmission between neurons, synaptic plasticity, and the overall stability of neural networks [35].

Recent studies have increasingly highlighted the role of GABRP in various tumors [36]. The GABA_A_ receptor, particularly the GABA_A_ receptor containing the π subunit (GABRP), is one of the most important inhibitory receptors in the central nervous system, regulating neuronal excitability and maintaining neurological balance [37]. It plays vital roles in controlling anxiety, influencing sleep, and modulating muscle activity. Emerging research indicates that GABRP is not only essential for central nervous system function but also significantly influences tumor initiation and progression, linking its roles in both areas [38]. Its functional diversity positions GABRP as a potential diagnostic biomarker and a promising therapeutic target in cancer treatment. By understanding the mechanisms by which GABRP influences the tumor microenvironment and immune evasion, we can pave the way for innovative strategies in cancer diagnosis and therapy, ultimately improving patient outcomes [39].

The immune system plays a crucial role in helping a healthy organism fight tumor cells by monitoring and responding to them [40]. The immune system identifies and eliminates abnormal or mutated cells to prevent the occurrence and progression of tumors [41]. Immune cells like T cells, natural killer (NK) cells, and macrophages recognize tumor‐specific antigens [42]. This recognition triggers immune responses, releases cytokines, and promotes attacks on tumor cells. Additionally, the immune system maintains the body's health balance by inhibiting tumor cell proliferation and metastasis . However, while a healthy organism has defense mechanisms, tumor cells often evade immune surveillance through various methods, such as reducing antigen expression, secreting immunosuppressive factors, or inducing immune tolerance. On one hand, the body can resist tumor formation through innate and adaptive immunity; on the other hand, tumor cells can evade immune recognition and attack through various mechanisms [43]. When the immune balance is disrupted, it creates an immunosuppressive environment that benefits tumor growth [44].

In our research findings, as shown, high expression of the GABRP gene in cancer cells affects tumor development, progression, and response to immunotherapy. GABRP copy number changes are associated with the infiltration of numerous immune cells, including regulatory T cells, B cells, CD8+ T cells, CD4+ T cells, neutrophils, and dendritic cells [45]. Notably, GABRP overexpression is positively correlated with regulatory T cells, M2 macrophages, and B cells, which are immunosuppressive cells. Therefore, GABRP overexpression can promote the accumulation of suppressive immune cells, leading to immune evasion by tumor cells across various cancers.

Our findings indicate that in various cancers, tumor cells increase GABRP gene expression, which alters the tumor microenvironment and leads to an excess of suppressive immune cells, facilitating immune evasion [46]. Why is it that only the GABRP gene regulates the complex tumor microenvironment in pan‐cancer? This is because only the GABRP gene in the human body can express GABA, and GABA is the primary inhibitory receptor in the body [47]. Besides regulating neuronal excitability, GABA also inhibits immune cells to modulate the tumor microenvironment [48].

The increased expression of the GABRP gene is crucial for tumor cells to evade immune surveillance, as it leads to a higher release of GABA [49]. This directly inhibits the growth and activity of nearby T cells, which contributes to immune evasion and tumor growth. Tumor cells control the release of GABA via GABRP, which changes the composition of immune cells in the tumor microenvironment. This promotes the accumulation of suppressive immune cells, such as M2 macrophages and regulatory T cells, further inhibiting anti‐tumor immune responses [50]. Furthermore, the upregulation of GABRP can impair dendritic cell function by reducing their antigen‐presenting ability. This ultimately weakens T cell activation [51].

Excessive GABA inhibits T cell proliferation by activating GABA_A_ receptors [52]. T cells express both GABA_A_ and GABA_B_ receptors and when GABA binds to these receptors, corresponding signaling pathways are activated, leading to intracellular signaling changes that suppress T cell activity [53]. GABA_A_ receptors are ion channel receptors, and their activation leads to the influx of chloride ions (Cl^−^) into the cell [54]. This chloride influx causes cell membrane depolarization, reducing cellular excitability, thereby inhibiting T cell proliferation and cytokine secretion. This mechanism is particularly important in the tumor microenvironment, as it leads to the overall suppression of T cell function [55].

Moreover, GABA notably affects other immune cells, particularly by shifting macrophage polarization toward the M2 phenotype [56]. This M2 polarization is associated with tumor‐related immunosuppression [57]. M2 macrophages further suppress T cell activity by secreting inhibitory factors. In addition, GABA can impair the function of dendritic cells by reducing their antigen‐presenting capabilities, thereby affecting T cell activation and function [58]. GABRP is often overexpressed in pan‐cancer. In the tumor microenvironment, the high expression of GABRP specifically inhibits the proliferation and activity of immune cells [59]. The elevated levels of GABRP function as a “protective barrier” for pan‐cancer tumor tissues, effectively preventing recognition and clearance by normal immune cells [60]. Additionally, the overexpression of GABRP acts as a “silencer,” negatively regulating neural impulses and immune cell activity, thereby promoting immune evasion in pan‐cancer [61].

Currently, cancer treatments have developed effective immunotherapies that utilize the patient's immune system to identify and attack cancer cells. This treatment approach mainly includes immune checkpoint inhibitors, CAR‐T cell therapy, monoclonal antibodies, and vaccines [62]. While immunotherapy is promising, it has challenges and limitations, including limited patient responses, potential severe immune‐related side effects (like autoimmune reactions), and high treatment costs [63].

According to our research findings, GABRP is a key oncogene that promotes tumor invasion and cell migration in cancer, and the use of Amentoflavone can effectively treat tumors. Therefore, the first approach for cancer treatment is to knock out the expression of the GABRP gene in tumor cells to release GABA. The second approach is to use GABA receptor inhibitors, such as Amentoflavone, continuously in the tumor microenvironment, making this a relatively simple and effective treatment method.

Recent research indicates that Mediratta et al. conducted in vitro and in vivo experiments demonstrating that GABA receptor inhibitors, such as Amentoflavone, play a crucial role in targeting TNBC [64]. Yen et al. conducted in vitro experiments on glioma cells and verified that flavonoid drugs can not only induce both endogenous and exogenous apoptosis in glioma cells but also reduce NF‐ĸB‐regulated anti‐apoptotic signals in vitro [65]. The two researchers successfully conducted treatment studies on certain cancers using Amentoflavone, but they were unaware of the key mechanisms behind the anti‐cancer treatment. These researchers also did not know how to correctly use GABA receptor inhibitors for anti‐cancer treatment; they only used them briefly in experiments, resulting in less effective outcomes.

**FIGURE 10:**
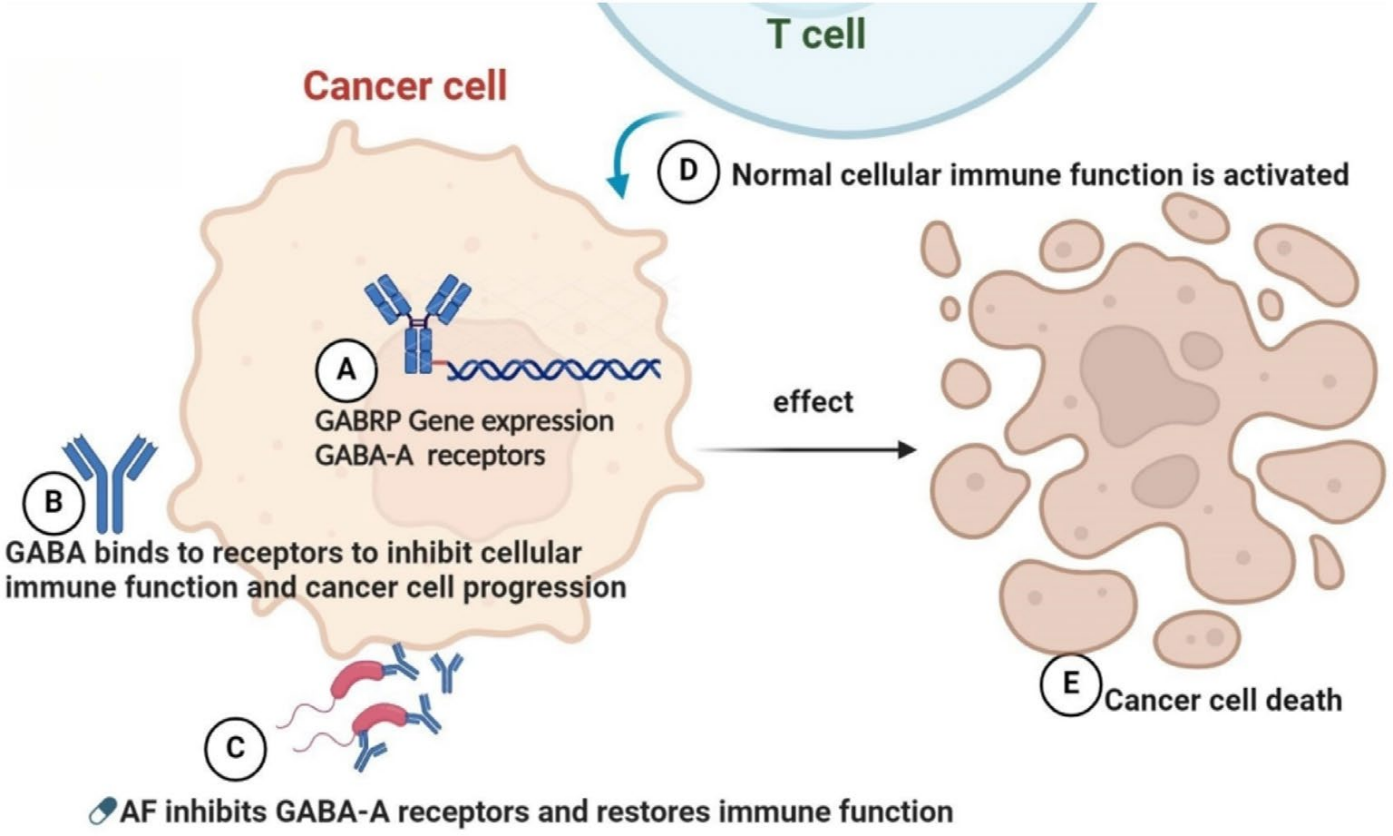
GABA overexpressed by cancer cells is inhibited by AF, restoring the normal immune function of the body to kill cancer cells (A) to (E).

This occurs because cancer cells can continuously produce excessive GABA by upregulating GABRP gene expression, which affects the tumor microenvironment. This necessitates the continuous suppression of GABA release or the use of GABA receptor inhibitors at the tumor growth site, and treatment measures can only be withdrawn after the tumor is eliminated [66]. Literature has confirmed the role of GABA receptor inhibitors in cancer treatment, and related drugs can indeed be found on MCE (Med Chem Express) that have therapeutic effects on cancer [67]. This evidence is sufficient to demonstrate that GABA receptor inhibitors, such as flavonoid drugs, have therapeutic effects on cancer, which also proves that the results of this study are scientific, authentic, and correct [68] (Figure 10).

As a member of the γ‐aminobutyric acid (GABA) receptor family, the association between GABRP's cancer‐promoting mechanism and tumor microenvironment remodeling provides a new direction for targeted intervention [69]. First, GABA receptor inhibitors (such as bicuculline and biculin) may inhibit tumor progression by blocking GABRP‐mediated signal transduction (such as PI3K/AKT or STAT3 pathways) [70]. Studies have shown that GABAergic signals promote cell migration in gliomas by activating chloride channels, and targeted inhibition of GABRP may reverse this effect and weaken its ability to recruit regulatory T cells (Tregs) and M2 macrophages, thereby relieving immunosuppression. Secondly, the natural compound amentoflavone has attracted much attention due to its multi‐target properties: on the one hand, it can inhibit downstream STAT3 phosphorylation by directly binding to GABRP, block IL‐10/CCL22 secretion, and reduce Tregs infiltration [71]; on the other hand, it can also synergistically enhance the efficacy of PD‐L1 inhibitors and may restore anti‐tumor immune responses by regulating T cell exhaustion markers [72]. However, challenges still exist: There are many subtypes of the GABA receptor family, and inhibitor selectivity needs to be optimized to avoid central nervous system side effects; Tumor heterogeneity may lead to differences in GABRP dependence, and biomarkers (such as GABRP^+^/PD‐L1^+^ double‐positive subgroups) need to be developed to screen the beneficiary population; The bioavailability and delivery efficiency of amentoflavone need to be improved (such as nanoliposome encapsulation). Future research should focus on combination therapy design (such as GABA inhibitors + immune checkpoint blockade) and preclinical pharmacodynamic validation, while using organoid models to evaluate response differences across cancer types to accelerate translational applications [73].

## 5 Conclusions

In summary, our research shows that the primary target of immune‐suppressive tumor microenvironments in pan‐cancer is the GABRP gene. Cancer cells upregulate this gene, resulting in an overproduction of suppressive immune cells that help tumors evade immune detection. To treat cancer, we can either continuously inhibit GABA release at local tumor sites in pan‐cancer or use GABA receptor inhibitors that can be discontinued after tumor elimination. Then, an effective method to treat cancer is to continuously inhibit the expression of the GABRP gene or use GABA receptor inhibitors in the growth sites of human cancer cells, which may produce a certain immune targeted treatment effect on cancer cells. After the tumor is eliminated, the treatment measures can be withdrawn. However, the study has its limitations, such as the small sample size and the potential lack of robust statistical significance of specific results. Our research has limitations, as it has yet to be validated through additional basic and clinical experiments. We believe that in our future research, we will gradually conduct more cell experiments, animal experiments, and clinical trials to study and eradicate cancer as soon as possible.

## Author Contributions

**Wu Cen:** conceptualization (lead), writing – original draft (lead). **Genyuan Fu:** methodology (equal), writing – review and editing (equal). **Ruting Wei:** formal analysis (equal), supervision (equal). **Xingwang Zhou:** funding acquisition (equal), validation (equal). **Wei Teng:** investigation (equal), visualization (equal). **Yuanguo Ling:** investigation (equal), writing – review and editing (equal). **Xiaoyu Wang:** data curation (equal), software (equal). **Jiaze Tang:** project administration (equal), writing – review and editing (equal). **Zhongan Wang:** methodology (equal), project administration (equal), writing – review and editing (equal). **Liangzhao Chu:** investigation (equal), methodology (equal), project administration (equal), writing – review and editing (equal).

## Conflicts of Interest

The authors declare no conflicts of interest.

## Supporting information


Figures S1–S10.

Table S2.

Table S3.

## Data Availability

GEPIA2 (11) (https://gepia2.cancer‐pku.cn/). The Cancer Genome Atlas (TCGA) (https://gdc.cancer.gov/). Human Protein Atlas (HPA) Analysis. HPA (14) (https://www.proteinatlas.org/). The Kaplan‐Meier plotter (17) (https://kmplot.com). cBioPortal (18) (https://www.cbioportal.org/). The original contributions presented in the study are included in the article/Figures S1–S10 and Table 1, Tables S2 and S3. Further inquiries can be directed to the corresponding author.
