# Supplementary material for: GABRP Mediates GABA‐A Receptor to Shape Tumor Immunosuppressive Microenvironment and Promote Tumor Immune Escape and Corresponding Targeted Therapy
Source: Cancer Med. 2025 Jun 3;14(11):e70946. doi: 10.1002/cam4.70946 (PMC12131280; doi:10.1002/cam4.70946)
Supplement: Supplementary file 1 — Figures S1–S10. [file CAM4-14-e70946-s003.pptx]

## Slide 1
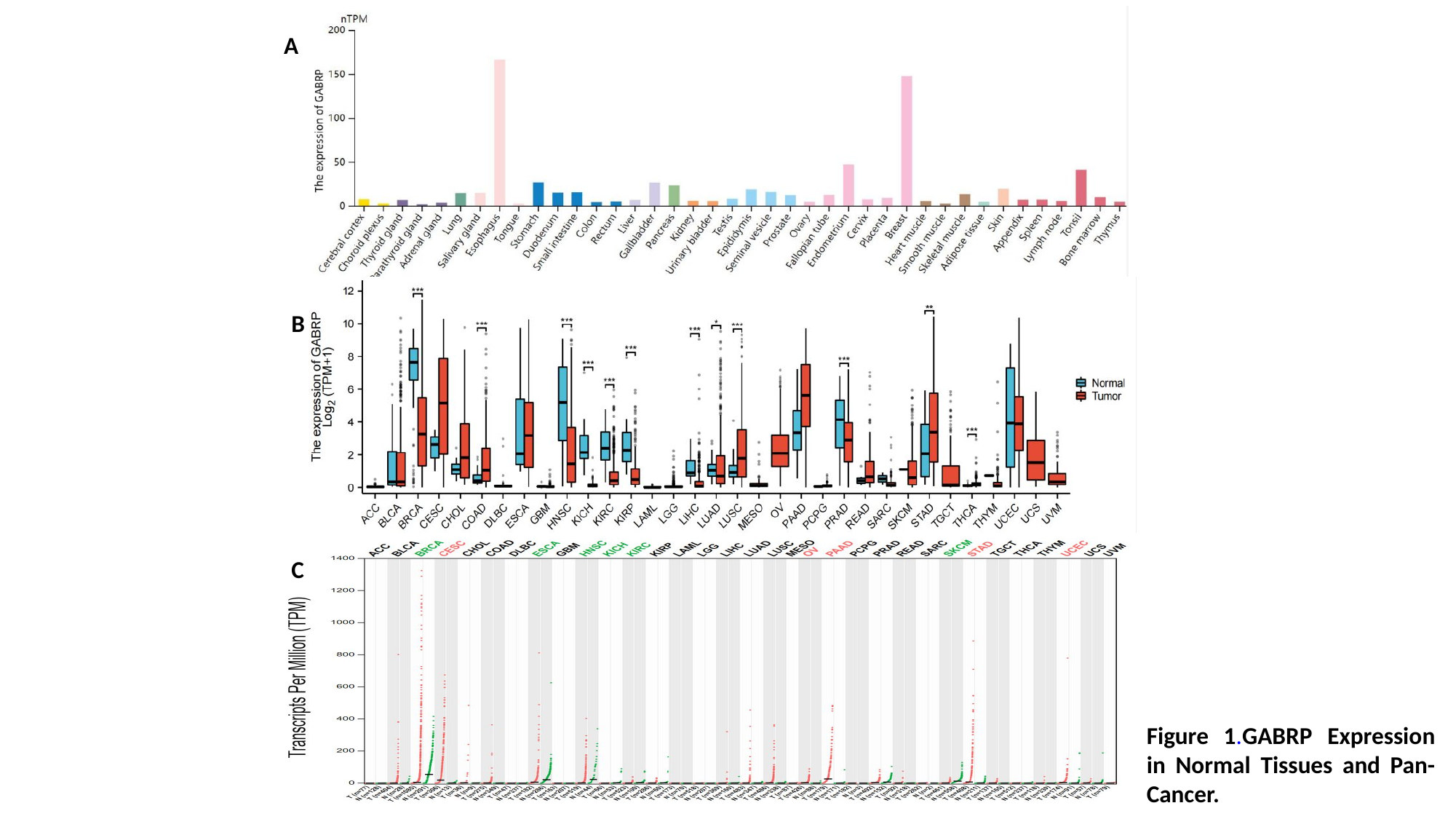

A
B
C
Figure 1.GABRP Expression in Normal Tissues and Pan-Cancer.

## Slide 2
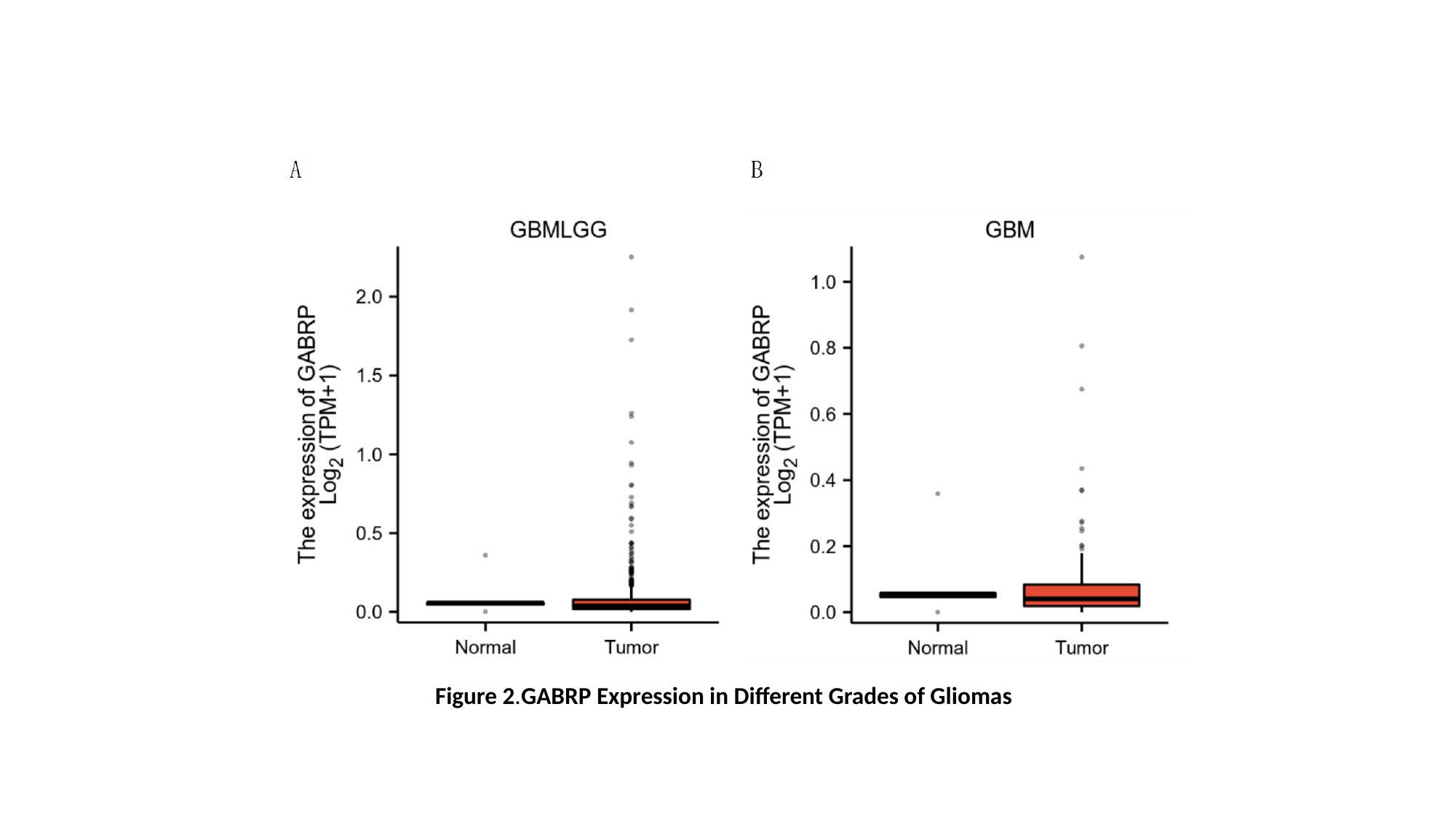

Figure 2.GABRP Expression in Different Grades of Gliomas

## Slide 3
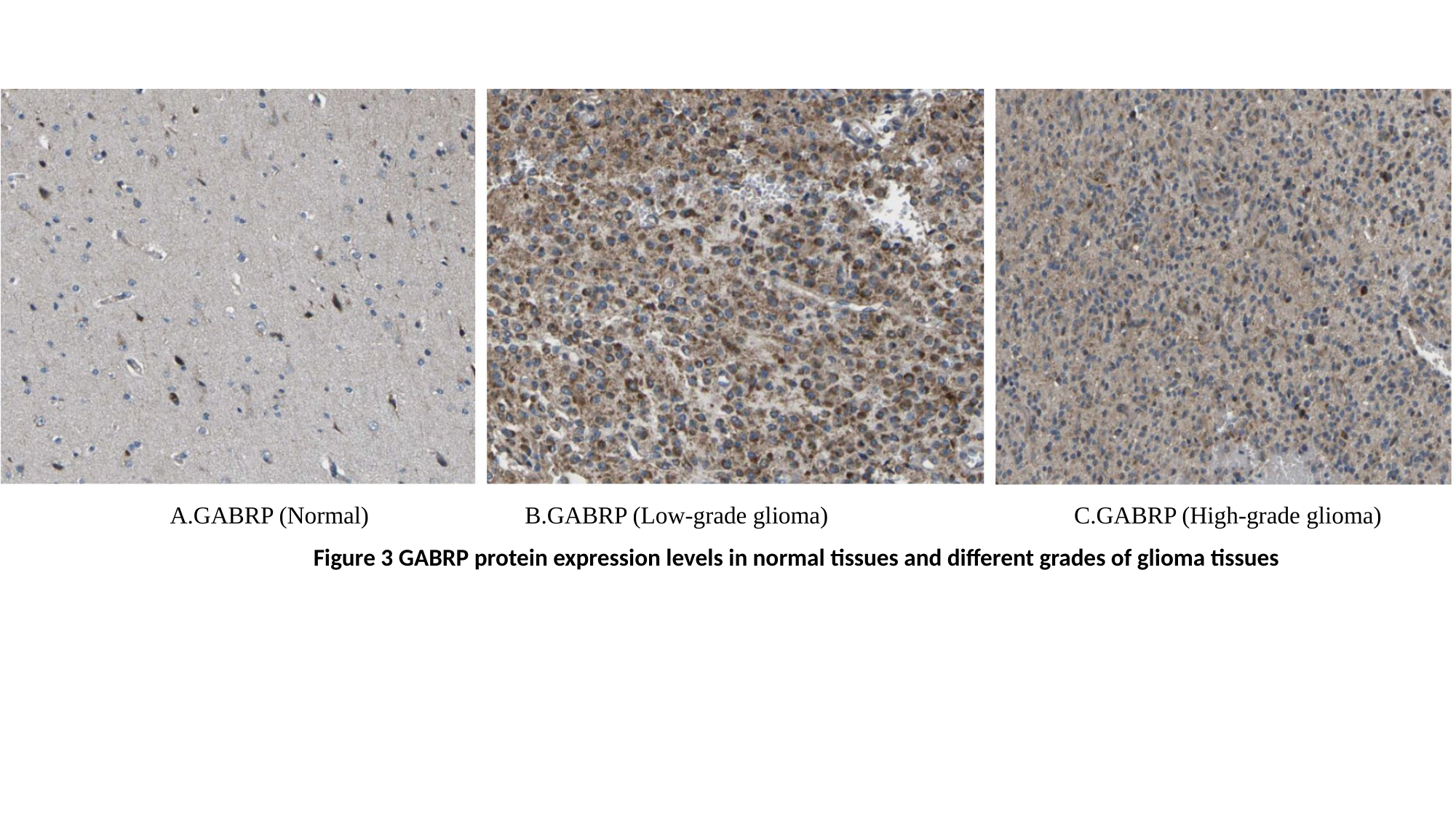

A.GABRP (Normal) B.GABRP (Low-grade glioma) C.GABRP (High-grade glioma)
Figure 3 GABRP protein expression levels in normal tissues and different grades of glioma tissues

## Slide 4
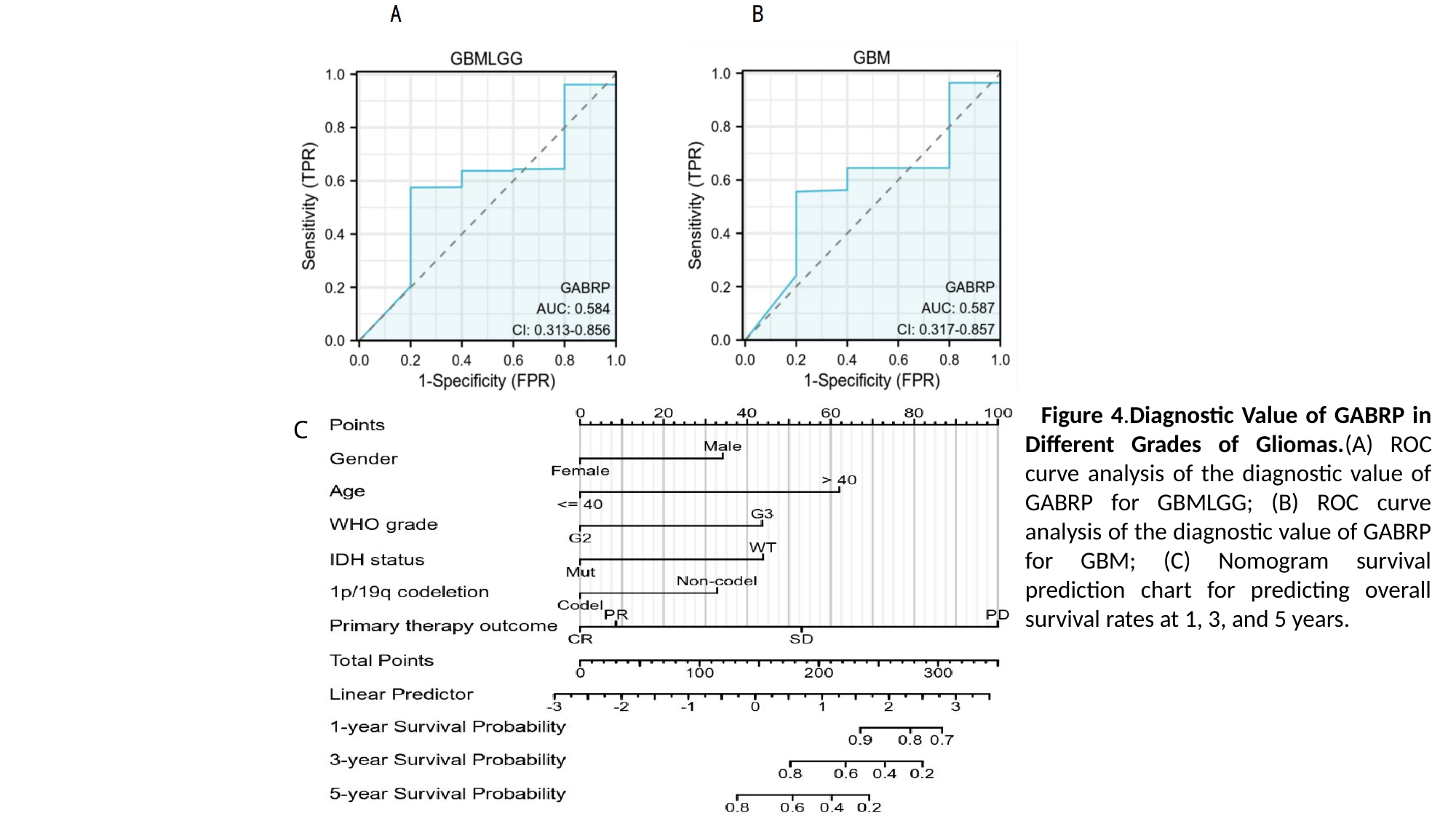

Figure 4.Diagnostic Value of GABRP in Different Grades of Gliomas.(A) ROC curve analysis of the diagnostic value of GABRP for GBMLGG; (B) ROC curve analysis of the diagnostic value of GABRP for GBM; (C) Nomogram survival prediction chart for predicting overall survival rates at 1, 3, and 5 years.
C

## Slide 5
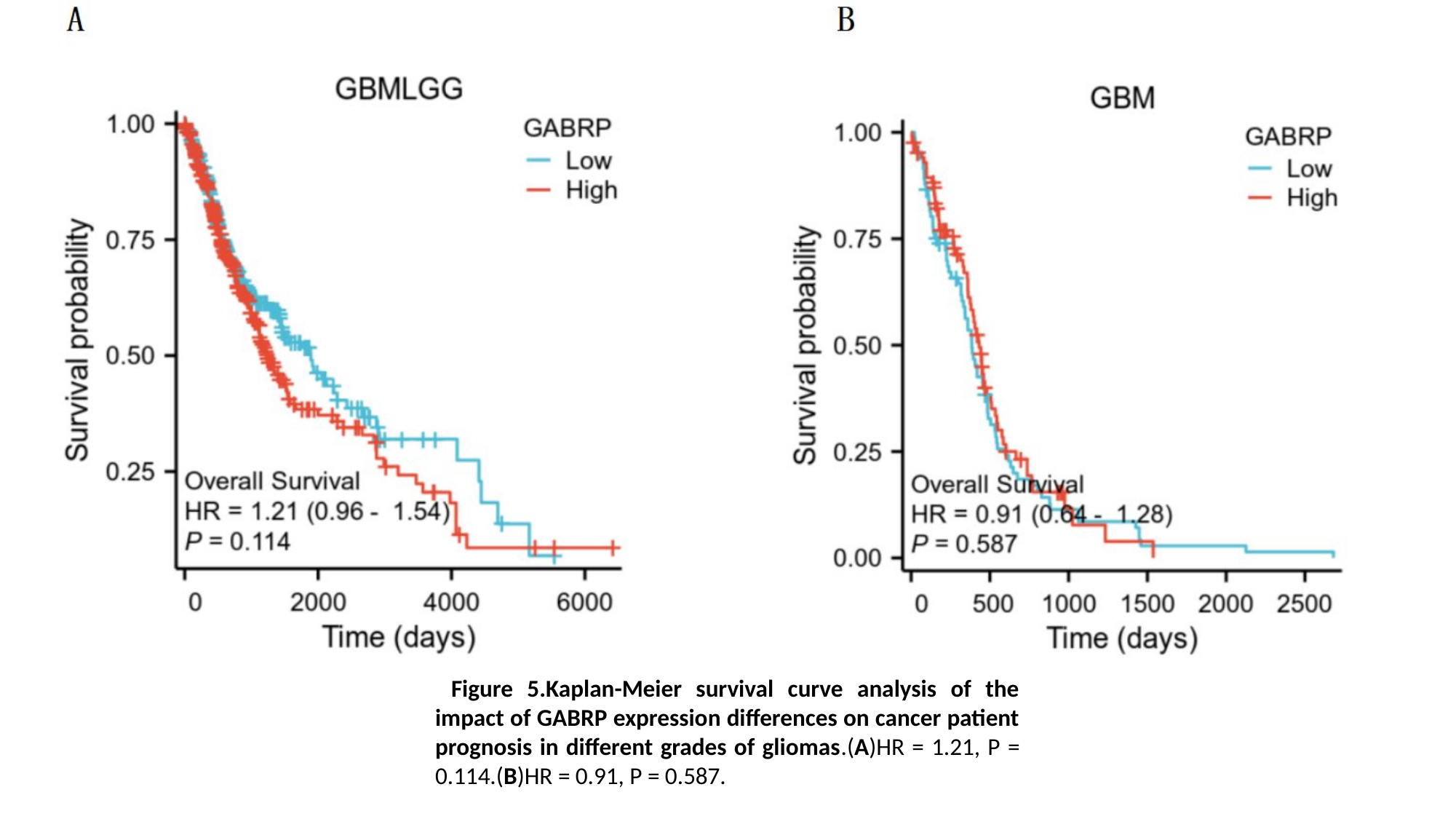

Figure 5.Kaplan-Meier survival curve analysis of the impact of GABRP expression differences on cancer patient prognosis in different grades of gliomas.(A)HR = 1.21, P = 0.114.(B)HR = 0.91, P = 0.587.

## Slide 6
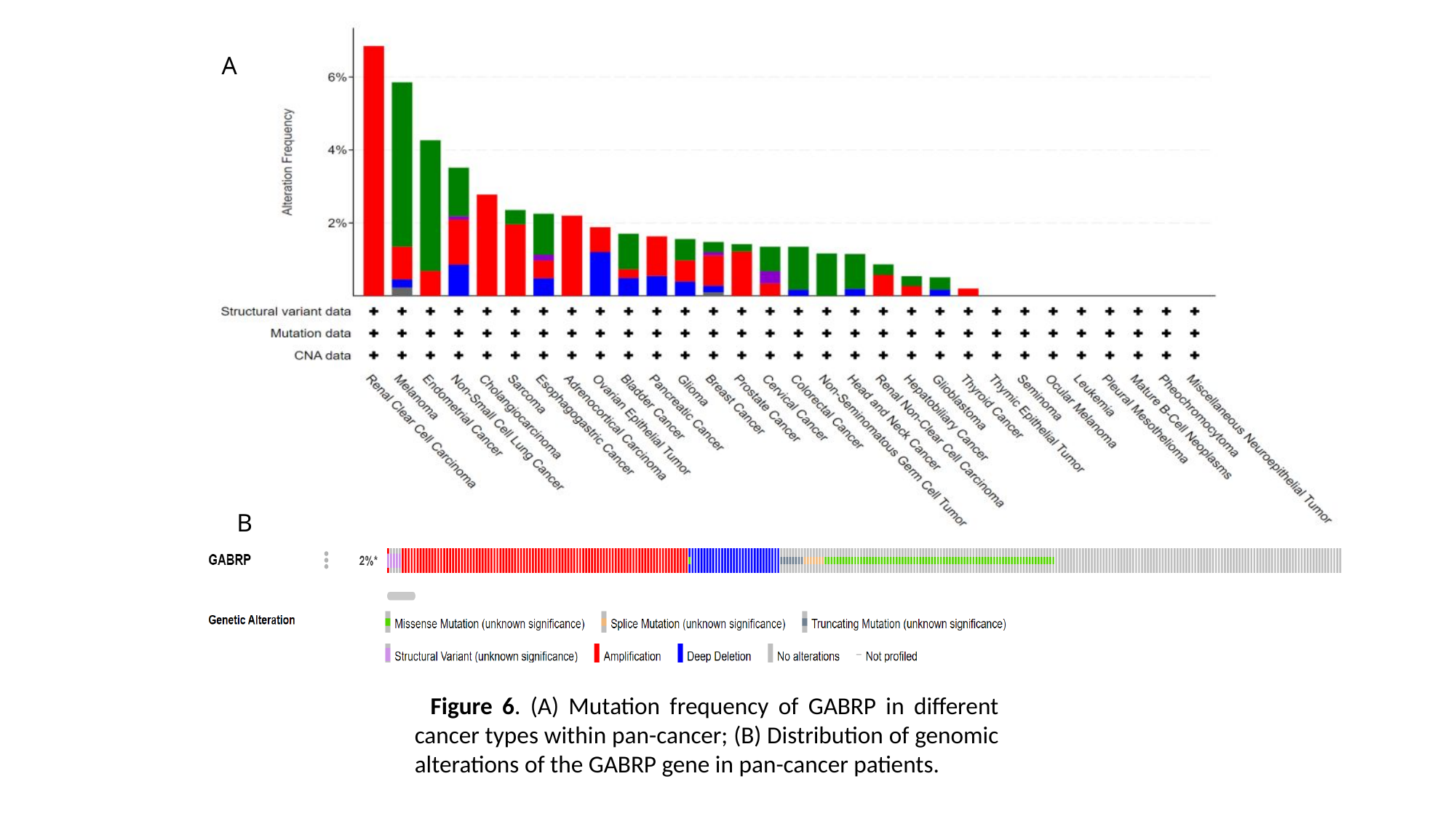

A
B
Figure 6. (A) Mutation frequency of GABRP in different cancer types within pan-cancer; (B) Distribution of genomic alterations of the GABRP gene in pan-cancer patients.

## Slide 7
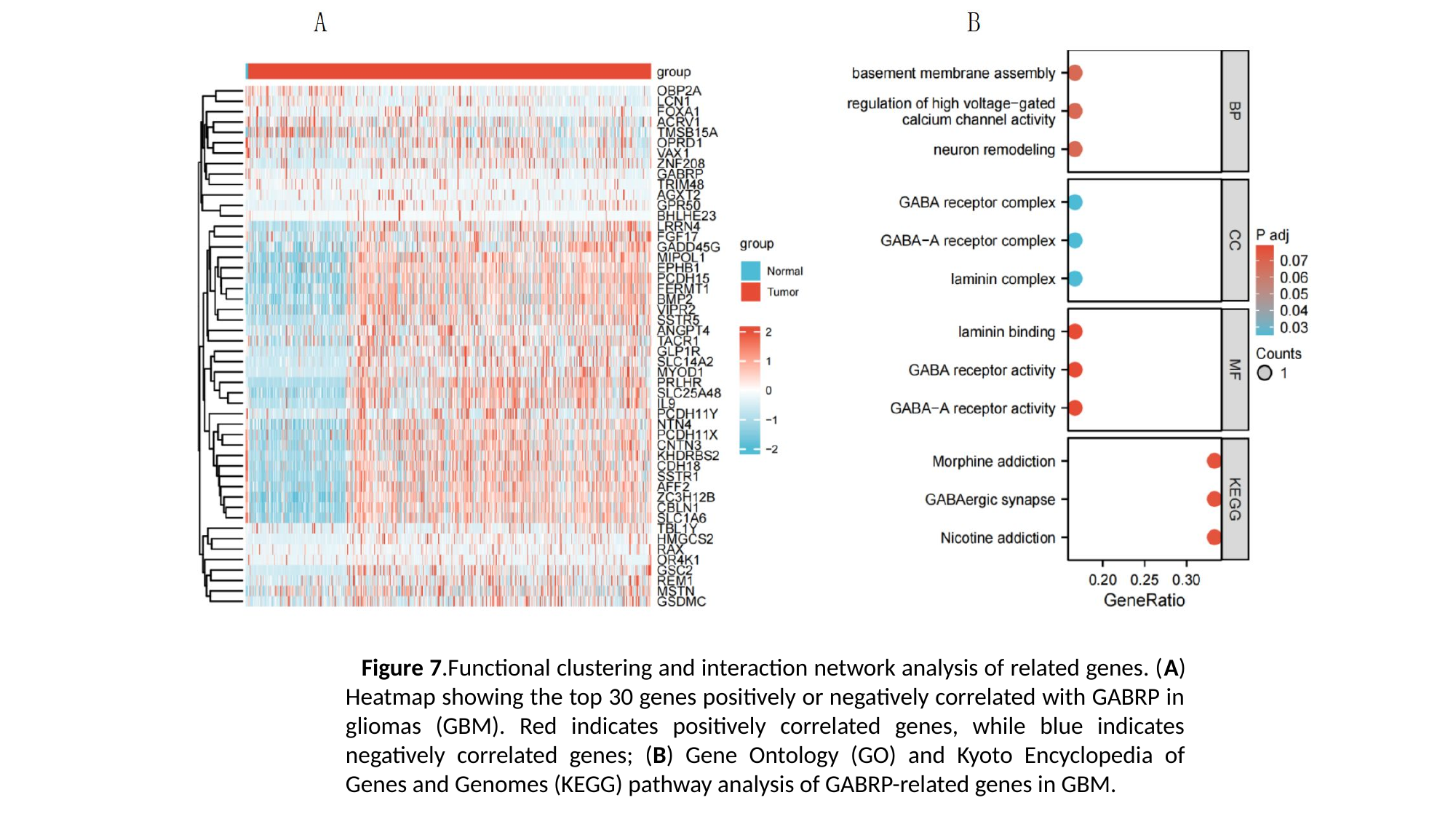

Figure 7.Functional clustering and interaction network analysis of related genes. (A) Heatmap showing the top 30 genes positively or negatively correlated with GABRP in gliomas (GBM). Red indicates positively correlated genes, while blue indicates negatively correlated genes; (B) Gene Ontology (GO) and Kyoto Encyclopedia of Genes and Genomes (KEGG) pathway analysis of GABRP-related genes in GBM.

## Slide 8
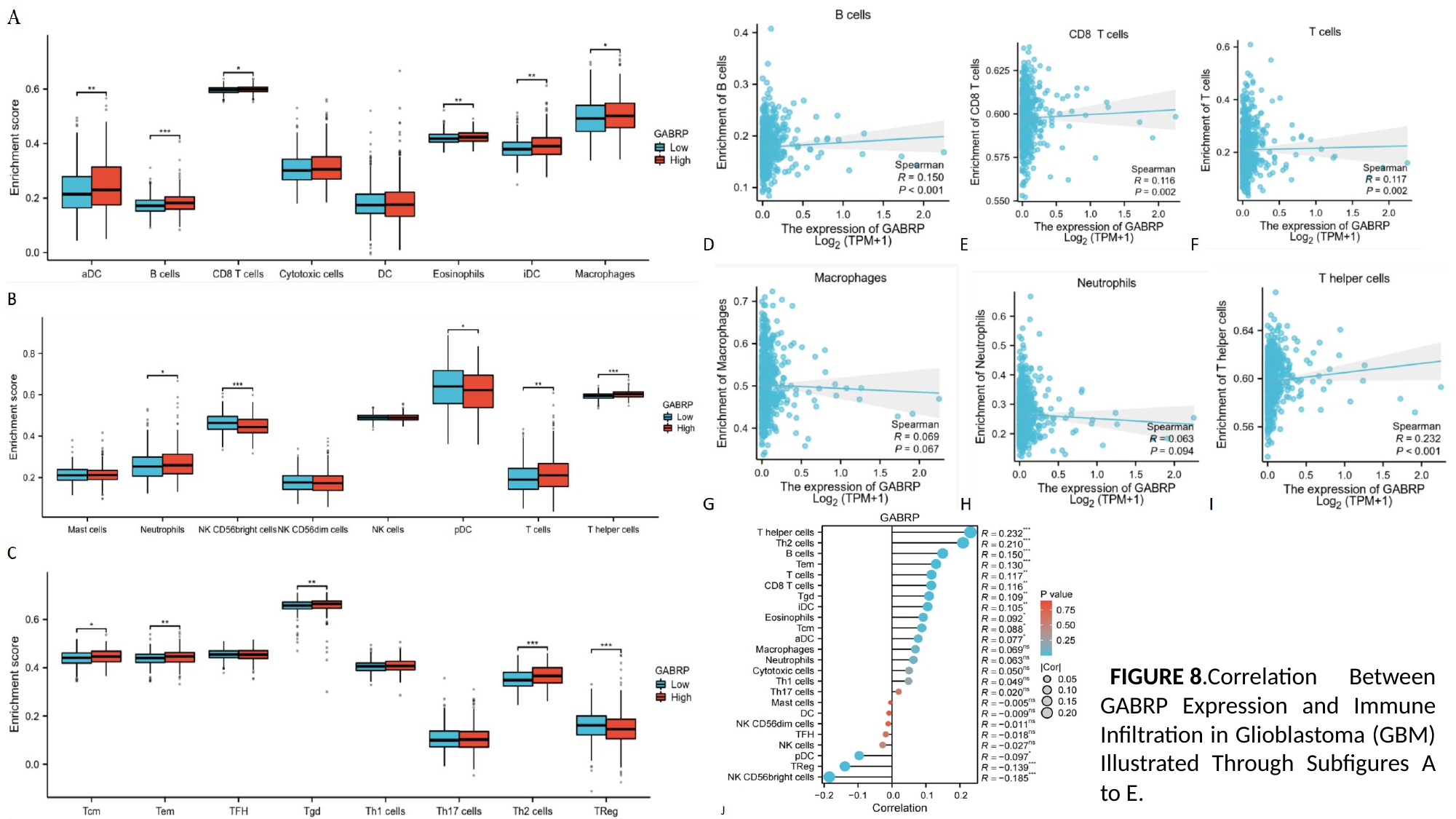

Figure 8.Correlation Between GABRP Expression and Immune Infiltration in Glioblastoma (GBM) Illustrated Through Subfigures A to E.

## Slide 9
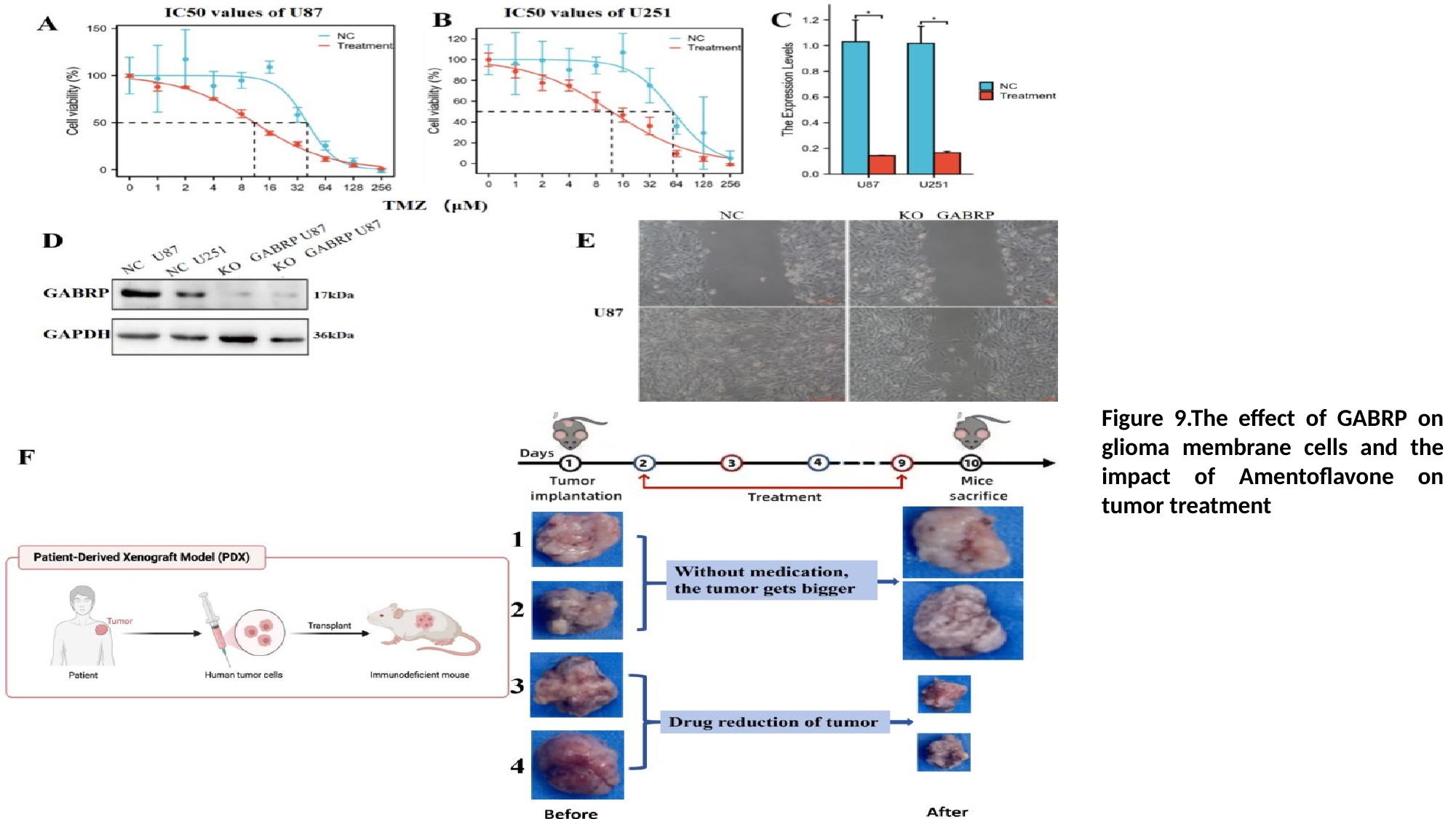

Figure 9.The effect of GABRP on glioma membrane cells and the impact of Amentoflavone on tumor treatment

## Slide 10
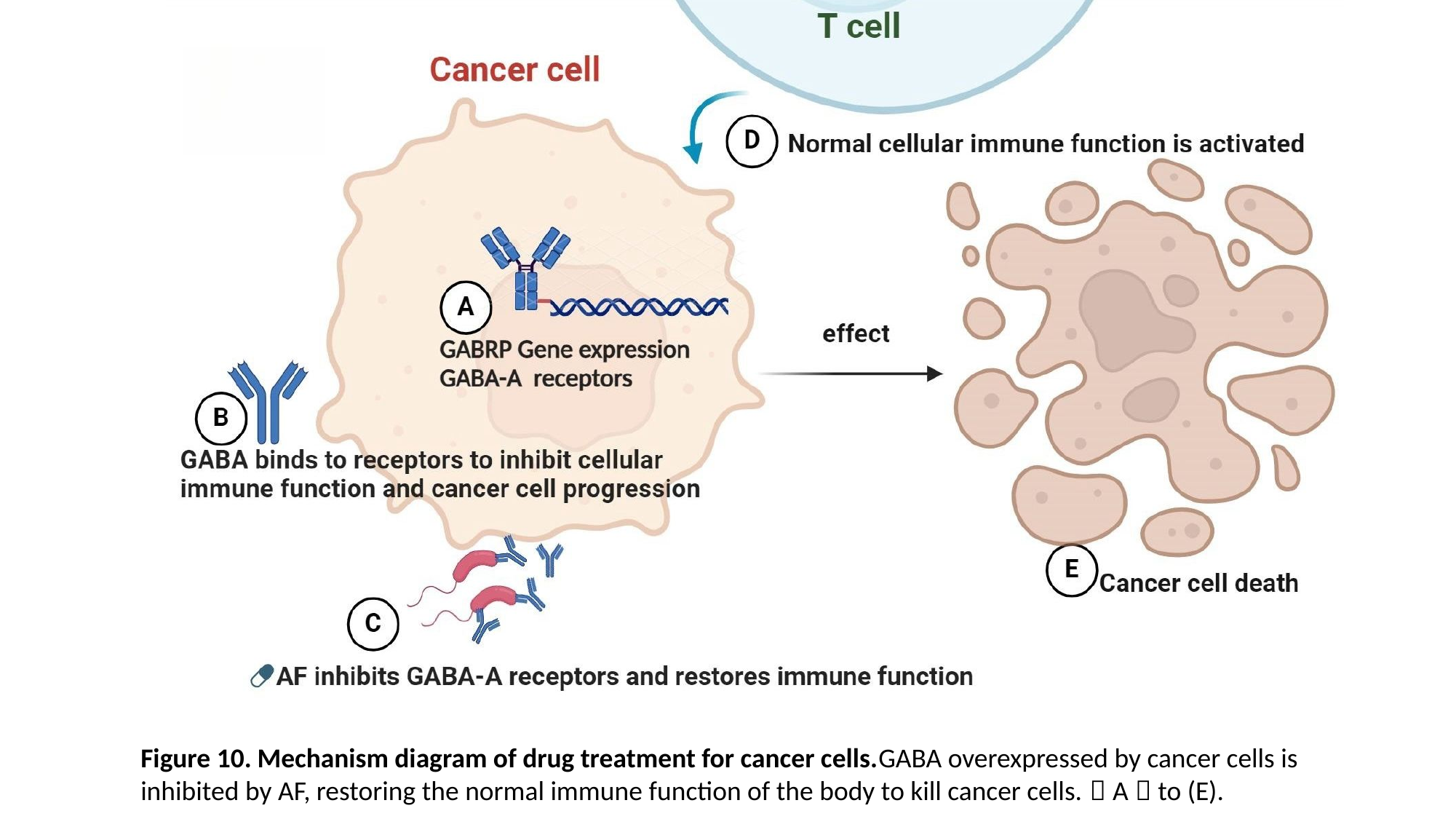

Figure 10. Mechanism diagram of drug treatment for cancer cells.GABA overexpressed by cancer cells is inhibited by AF, restoring the normal immune function of the body to kill cancer cells.（A）to (E).
